# Predicting school students’ physical activity intentions in leisure-time and school recess contexts: Testing an integrated model based on self-determination theory and theory of planned behavior

**DOI:** 10.1371/journal.pone.0249019

**Published:** 2021-03-26

**Authors:** Heidi Pasi, Taru Lintunen, Esko Leskinen, Martin S. Hagger

**Affiliations:** 1 Faculty of Sport and Health Sciences, University of Jyväskylä, Jyväskylä, Finland; 2 Department of Mathematics and Statistics, University of Jyväskylä, Jyväskylä, Finland; 3 Psychological Sciences, University of California, Merced, California, United States of America; La Inmaculada Teacher Training Centre (University of Granada), SPAIN

## Abstract

**Background:**

Identifying psychological correlates of children’s physical activity intentions may signpost potentially modifiable targets for interventions aimed at promoting physical activity participation. School recess and leisure-time outside of school are appropriate contexts in which such interventions may be delivered. However, few studies have identified correlates of physical activity intentions in these environments. Examining correlates in these contexts may provide formative evidence on which to base interventions to promote physical activity.

**Purpose:**

The current study adopted an integrated theoretical model to test relations between motivational constructs from self-determination theory, social cognition constructs from the theory of planned behavior, and physical activity intentions in leisure-time and school recess contexts.

**Methods:**

Finnish school children (*N* = 845, *M* age = 13.93, *SD* = 0.99) from three lower-secondary schools completed self-report measures of perceived autonomy support by peers, autonomous and controlled motivation, attitudes, subjective norms, perceived behavioral control, and physical activity intentions for both contexts.

**Results:**

Well-fitting structural equation models controlling for past behavior indicated that autonomous motivation in the school recess context and attitude in both contexts were the most pervasive predictors of physical activity intentions, and mediated the relationship between perceived autonomy support and intentions. Multi-group analyses supported invariance of the models in both contexts across gender, grades, and school, with few variations.

**Conclusions:**

The current study supports relations between motivational and social cognition correlates of children’s physical activity intentions in school recess and leisure-time contexts. Future research should extend these findings to the prediction of follow-up participation in physical activity.

## Introduction

Participation in regular moderate-to-vigorous physical activity in childhood and adolescence confers multiple short- [1] and long-term [2] benefits. Consequently, national and international physical activity guidelines stipulate that children and adolescents should engage in moderate-to-vigorous intensity physical activity, that includes aerobic and strength components, for at least 60 minutes per day [3,4]. However, few children meet these guidelines, and rates of children meeting guideline levels of physical activity decline throughout adolescence [5,6]. Promotion of physical activity in young people through intervention has therefore been identified as a priority by governments and health promotion organizations [7,8].

Behavioral scientists and health promotion practitioners have advocated the imperative of identifying theoretical determinants of physical activity behavior that should be targeted in interventions [9,10]. Much of this research has focused on the determinants of leisure-time physical activity [11] or physical education (PE) [12]. The heavy focus on these contexts has been because leisure-time is context in which children are most likely to have sufficient time to fulfil activity guidelines, and PE is a context in which teachers have high access to the child population and can, therefore, promote messages and skills that can promote physical activity outside of school [13].

However, promoting physical activity in recess has also been identified as an appropriate existing context in which to promote physical activity in children [14], not only because it provides health promoters and interventionists with high access to school children, but also because it accounts for considerable proportion of school children’s time during school day–time which could be dedicated to increased participation in regular physical activity toward meeting guidelines [14,15]. Duration of recess periods varies across grades and national education systems, with a reported average of 49 minutes per day across 20 countries [15]. In Finland, for example, students enter lower secondary school in August on that calendar year when they turn thirteen years and spend an average of 60 to 70 minutes per day in recess during the three years they spend at this school stage (grades 7, 8 and 9) [16,17]. Recess periods, therefore, offer plenty of scope for children to participate in physical activity during school and to make a substantive contribution to meeting activity guidelines [14]. However, only a portion of lower secondary school students are physically active during recess [17,18]. For example, 70% of lower secondary school students reported that they never participate in physically active play during recess [17]. In addition, physical activity participation during recess declines from primary school to lower secondary school [18,19]. Given that school recess is a context which offers considerable potential in terms of the time that could be dedicated to physical activity, identifying the modifiable psychological correlates of school students’ physical activity participation may have utility in informing efforts to promote physical activity.

In the current study, we examined the motivational and social cognition antecedents of school students’ intentions to participate physical activity in two contexts: leisure-time and school recess. Comparison of the correlates of physical activity participation in different contexts is important because the determinants of physical activity in different contexts may not be considered identical [20]. For example, although both school recess and leisure-time reflect ‘free time’, the recess context differs in many ways to the leisure-time context (e.g., time available, physical and social environment). Comparison of correlates of physical activity in each context is important order to identify the most salient modifiable targets for interventions to promote physical activity in each context.

The current research is based on an integrated theoretical model drawing from two theories of motivation, the theory of planned behavior [21] and self-determination theory [22,23]. The theory of planned behavior is a social cognition theory that specifies the belief-based antecedents of intentional behavior [21]. According to the theory, intention is the most proximal determinant of behavior. Intention is a motivational construct that reflects the extent to which people are willing to try and plan to perform a target behavior in future. Intentions are a function of three belief-based constructs: attitudes reflect the positive or negative evaluation of performing the behavior in future, subjective norms reflect the perceived social pressure to participate in the behavior, and perceived behavioral control reflects beliefs in capacity to perform the behavior [21]. Meta-analyses have supported the predictions of the theory in health behaviors [24], including physical activity [11]. Importantly, intention is the most proximal predictor of physical activity behavior, and meta-analyses have demonstrated small-to-medium effect sizes for the intention-behavior relationship [11,25].

Self-determination theory is a needs-based theory of motivation [22,23]. The theory predicts that the quality or *type* of motivation is paramount in determining behavior, rather than quantity alone. Central to the theory is the distinction between autonomous and controlled forms of motivation. Autonomous forms of motivation reflect motives or reasons for participating in a given behavior that are perceived as chosen, personally-consistent, and endorsed by the individual’s true sense of self. Controlled forms of motivation reflect participation in behaviors for externally-referenced reasons such as obtaining rewards or gaining approval from others, or avoiding punishment or others’ disapproval. Autonomous and controlled forms of motivation are often viewed on a continuum reflecting relative level of self-determination [23]. Overall, research has demonstrated consistent effects of autonomous motivation on persistence with multiple health behaviors [26], including physical activity [27].

Given that autonomous forms of motivation are consistently linked to adaptive outcomes and behavioral persistence in physical activity, promoting autonomous motivation may assist in promoting behavior change and behavioral adherence. One means to promote autonomous motivation is through the interpersonal ‘climate’ fostered by social agents such as teachers, parents, and peers [28]. Peers, for example, can support autonomy by offering encouragement and positive feedback, supporting competence, and providing a rationale for class activities. Primary studies and meta-analyses have demonstrated that interventions that induce social agents to foster autonomy supportive interpersonal environments lead to greater autonomous motivation, behavioral persistence, and adaptive outcomes (e.g., positive affect, well-being) compared to interventions fostering neutral or controlling environments [26,29]. It is often difficult to directly measure interpersonal environment (e.g., by observing teachers’ autonomy-supportive and controlling behaviors), so researchers often use perceived autonomy support of students to tap the extent to which the learning environment teachers create support students’ autonomy. Perceived autonomy support by significant others has been consistently related to autonomous motivation in leisure-time and school PE [30].

Recent research has integrated the theories of planned behavior and self-determination theory in order to arrive at a comprehensive model that outlines the processes by which individuals’ beliefs and motives relate to behavior [e.g., 31–33]. Self-determination theory compliments the theory of planned behavior as it gives rationale on why people form beliefs and intentions to seek certain outcomes. Similarly, the theory of planned behavior describes the process by which forms of motivation, autonomous or controlling, lead to intentions and subsequent action. This is based on the premise that individuals strategically align their beliefs with respect to participating in behavior in the future with their motives in order to set in motion the process of enacting the behavior. Individuals that perceive a particular behavior to be autonomously motivated are more likely to develop positive attitudes toward performing the behavior, and believe that they have control over performing the behavior. Analogously, subjective norms generally reflect compliance with significant others (e.g., teachers, peers, or parents), and have, therefore, been proposed to be aligned with controlled motives to participate in behavior. Research integrating the two theories has supported the proposed effects [30–32].

Few studies have applied constructs from motivational and social cognition theories to predict physical activity in contexts outside school PE, such as during recess and lunch breaks [34–36]. For example, research has indicated that self-determined forms of motivation are related to participation in unsupervised physical activity in school PE [34], and physical activity participation during recess periods [35]. Research integrating self-determination theory and the theory of planned behavior indicate that attitude and perceived behavioral control mediated effects of autonomous motivation on intentions and participation in physical activities during lunchtime recess [36]. However, there is, to date, no research that has applied the integrated model to study physical activities during school recess, or compared the motivational and social cognition antecedents of secondary school students’ participation in physical activity between leisure-time and recess contexts. The current study aimed to address this gap in the literature by employing an integrated theoretical model to test and compare the effects of theory constructs in predicting intentions to participate in physical activity in two contexts in which adolescents have high opportunity to participate in physical activity: leisure-time and school recess.

### Aims and hypotheses

**Fig 1:**
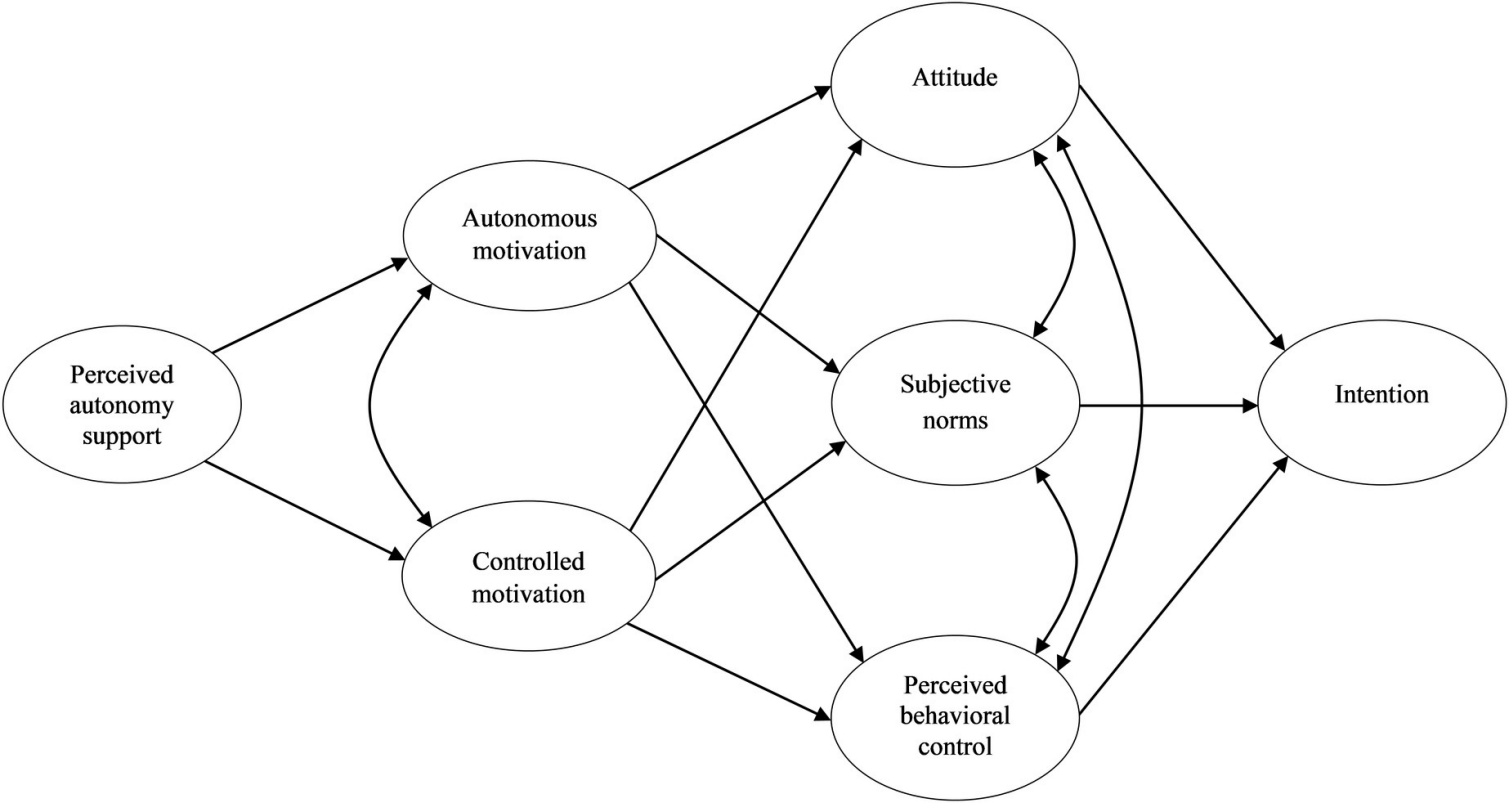
The hypothesized integrated model based on self-determination theory and the theory of planned behavior with perceived autonomy support from peers. Unidirectional arrows represent hypothesized paths; Bidirectional arrows represent estimated covariances between latent variables.

We aimed to investigate the theory-based antecedents of intentions to participate in physical activity during leisure-time and school recess among students from three lower secondary schools in Finland. We tested an integrated process model derived from self-determination theory and the theory of planned behavior. Specifically, we proposed that perceived autonomy support from students’ peers would be related to students’ autonomous and controlled motives to participate in physical activity in both contexts. In addition, autonomous and controlled motivation were hypothesized to mediate relationships between perceived autonomy support and the constructs from the theory of planned behavior and intentions. The hypothesized model is presented in Fig 1. We tested our model in leisure-time and recess contexts separately.

We also tested whether model predictions hold across gender, grade (grades 7 to 9), and school in the leisure-time and recess contexts. We proposed that the hypothesized paths in our process model represent generalized associations that apply across groups, consistent with the assumptions of the component theories of the model [21,22]. We therefore expected relations among model constructs in the models for both contexts to remain consistent (invariant) across these ecological and demographic factors. The sufficiency of the models in leisure-time and recess contexts was further tested by controlling for past physical activity, as recommended by theorists applying social cognition models to predict physical activity [21,31,37–40]. Finally, we tested the invariance of model parameters between leisure-time and recess contexts. As students are generally free to choose the activities they pursue during recess periods, we hypothesized that the recess context model will have the similar path structure to the leisure-time context model. However, we specified no direction in prediction–although we expected similar patterns of associations across the two contexts, we wanted to explore whether the relative contribution of model constructs (i.e., the size of the effects) differed across contexts.

## Methods

### Participants, design, and procedure

The study protocol was approved in advance by the Finnish Ministry of Education research review board (register number 76/627/2008). Native Finnish speaking students from three lower secondary schools in southern and central Finland were invited to participate in the study. The heads of the school districts and school principals provided consent for the data collection. Each participant was given an informed consent form to sign. In addition, pupils were provided with verbal information on the study requirements and were informed that participation was voluntary. The study adopted a single-wave correlational survey design. Members of the research team delivered the questionnaires in classrooms during normal school lessons and questionnaires were returned to the researchers after the lessons.

Of the 1293 students eligible to participate, 994 were contacted and consented to participate in the study (response rate 76.9%). We aimed to test our hypothesized model in leisure-time and recess contexts, so we only analyzed data of students who provided responses to study measures in both contexts. We therefore excluded data from 140 students resulting in a final sample size of 845 students aged between 12 to 16 years (females, *n* = 457; males, *n* = 388), including 292 seventh grade students (*M* age = 12.89, SD = .53), 259 eighth grade students (*M* age = 13.97, SD = .46) and 294 ninth grade students (*M* age = 14.94, SD = .50). Students were relatively evenly distributed across schools (School A, *n* = 325; School B, *n* = 274; School C, *n* = 246).

### Measures

Participants were asked to complete measures of the motivational and social cognition constructs from the proposed model with reference physical activity in both leisure-time and recess contexts. Items measuring the variables from self-determination theory and theory of planned behavior in leisure-time were derived from previous studies [30,41]. Measures were translated into Finnish following a back-translation procedure. These items were adapted to make reference the recess context. Examples of the measures and scales for leisure-time only are provided below and a full set of items and scales for both contexts are available as supporting information (S1 and S2 Tables). Prior to completing the questionnaires, participants were presented with a definition of what was meant by “physical activities” in the questionnaire: “By physical activities, we mean all the activities which make your heartbeat rise and makes you out of breath outside the school day” and “By physical activities, we mean all active physical activities at recess (e.g., brisk walking or ball games)” in leisure-time and recess contexts, respectively. We made reference to these examples because we wanted to rule out the casual low-paced walking and other low intensity activities in which students engaged at recess.

Intentions were measured on three items (e.g., “I intend to do physical activities, for at least 20 minutes at the time, over the next 4 weeks at the following regularity”). Responses were provided on 7-point scales (1 = not at all and 7 = everyday).

Attitudes were measured in response to a common stem: “Me doing physical activities for at least 20 minutes, 3 days per week over the next 4 weeks during my free time is. . .”, with responses provided on five 7-point semantic differential scales (e.g., 1 = *useless* and 7 = *important*).

Subjective norms were measured with three items (e.g., “Most people who are important to me would want me to do physical activities for at least 20 minutes, 3 days per week during my leisure-time over the next 4 weeks” with responses provided on 7-point scales (1 = *strongly disagree* and 7 = *strongly agree*).

Perceived behavioral control was measured on three items (e.g., “If I wanted to I could do physical activities for at least 20 minutes, 3 days per week during my free time over the next 4 weeks”), with responses provided on 7-point scales (1 = *strongly disagree* and 7 = *strongly agree*).

The behavioral regulations in exercise questionnaire [42] was used to measure autonomous and controlled motivation in leisure-time and recess contexts. Participants responded to a common question: “Why do you do physical activities in your leisure–time?” followed by 16 reasons representing the four motivational regulations from self-determination theory. Items measured intrinsic motivation (e.g., “Because it is fun”), identified motivation (e.g., “Because I value the benefits of physical activity”), introjected motivation (e.g., “Because I feel guilty when I don’t do physical activities”) and extrinsic motivation (e.g., “Because other people say I should”). Responses to each item were provided on 5-point scales (1 = not at all true and 5 = very true). We used item parceling to develop four indicators of autonomous and controlled motivation using a random selection procedure to minimize numbers of variables [43] (see S1 Appendix, supporting information for details).

Perceived autonomy support from peers for physical activity was measured on seven items (e.g., “My friends encourage me to do physical activities in my free time”) with responses provided on 7-point scales (1 = *strongly disagree* and 7 = *strongly agree*) [30,44]. Item parceling was applied to reduce number of items [43] (see S1 Appendix, supporting information for details).

Physical activity behavior in leisure-time was measured with two questions from the Finnish Health Behavior among School-Aged Children study [45] (e.g., “Over a typical or usual week, how often are you physically active, and sweat and get out of breath outside school hours?”).

Schools make the distinction between lunch breaks and shorter recess breaks between lessons. Therefore, physical activity at recess was measured with two questions, one related to lunch breaks and another related to regular breaks. (e.g., “During the last three weeks: On how many lunch breaks have you been doing physical activities at least half of the break time on average?”, responses with 6-point scale, 1 = *not at any lunch break* and 6 = *at all lunch breaks*).

### Data analytic strategy

Data were analysed using confirmatory factor analyses (CFA) and structural equation modelling using the Mplus software (version 8) [46]. All models were estimated using the robust maximum likelihood (MLR) estimator as it provides relatively stable estimates in data that is not normally distributed. Missing data were imputed using full-information maximum likelihood (FIML) imputation [46]. First, the structure of the measures was examined in separate CFAs in the full sample for each latent factor. Second, the CFAs were run separately in each gender (males, females), grade (grades 7 to 9), and school (School A, School B, School C) subgroup and subjected to an invariance routine testing the invariance of the configural model followed by the introduction of constraints on the factor loadings. We did not test invariance of factor variances and error variances as this was deemed too stringent [47].

The next step was to test the proposed model (see Fig 1) using structural equation model (SEM) in the full sample, followed by tests of invariance of measurement parameters across gender, grade, and school subgroups. In the event that the specification of the configural structural equation models for the subgroups involved modification of covariances between the observed items or item parcels, the measurement invariance of the factors was subsequently re-tested after modifications.

In a final phase, the structural invariance of model paths was tested by imposing equality constraints on proposed paths among factors across subgroups. In addition, the sustainability of the hypothesized theoretical paths was tested by including past physical activity as a covariate in the full sample model. All the analytic phases described above were conducted separately for the leisure-time and recess contexts. In the final phase of the analysis, comparisons of the size of the estimated paths in the leisure-time and recess contexts for the whole sample models were made using confidence intervals about the parameter estimates.

Model fit was assessed using multiple fit indices: the Yuan-Bentler scaled chi-square value of model fit (YBχ^2^), the comparative fit index (CFI), and the root mean square error of approximation (RMSEA). Values of .92 or greater for CFI and .07 or less for RMSEA were indicated acceptable model fit [48]. Differences in sets of parameters including factor loadings and model pathways across gender, grade, and school were tested using multigroup analysis. Multigroup models were evaluated based on incremental change in comparative fit index (ΔCFI) and on incremental change in Satorra-Bentler scaled chi-square value (ΔSBχ^2^) between the constrained and unconstrained models. Sets of parameters in the invariance routine were considered non-invariant if ΔCFI was > .01 [49] or if there was statistically significant ΔSBχ^2^ [48] together with modification indices above 10 for the non-invariant parameters. Model integrity was also assessed using model solution estimates: factor loadings, path estimates, composite reliabilities and average variance extracted. Data files, Mplus analysis scripts, and output files are available online at: https://osf.io/rtkmh/.

## Results

### Factor analytic models

The construct validity of the measures was good as the CFAs exhibited satisfactory fit and factor loadings above .40 in all cases with one exception. One item measuring subjective norms (“Most people who are important to me approve me to do. . .”) had low factor loadings in the full sample model and across sub-groups in both contexts. Consequently, this item was removed from the final SEM analysis. In terms of item content, the excluded item measured peer approval whereas all of the other subjective norms items measured expectations of significant others. Before deleting the items, the composite reliability of the subjective norm scale was .73 for both contexts. Otherwise, the composite reliabilities of the scales were between .80 and .93. Average variance extracted scores were between .48 and .82. Descriptive statistics, factor correlations of latent variables, composite reliabilities, and average variance extracted values for all items in the full sample measurement models for both contexts are provided in S3 Table in supporting information materials.

Results of the multigroup CFAs used to independent test the factor structure and invariance of each latent factor across gender, grade, and school provide support for invariance with only minor modifications. None of the loadings of the observed or parceled items was consistently non-invariant across any multigroup analyses for gender, grade, and school in either context. Full measurement invariance was supported for most factors across multigroup CFAs with the exception of five factors, for which partial measurement invariance was supported. Based on ΔSBχ^2^ and ΔCFI, factor loadings for one intention and one perceived autonomy support item were non-invariant across schools in the leisure-time context. One attitude item and one perceived behavioral control item was non-invariant across gender, and across grades and schools, respectively, in the recess context. Goodness-of-fit indexes of the multigroup CFAs and invariant factor loadings are provided in S4 Table in supporting information materials.

### Structural equation models

**Fig 2:**
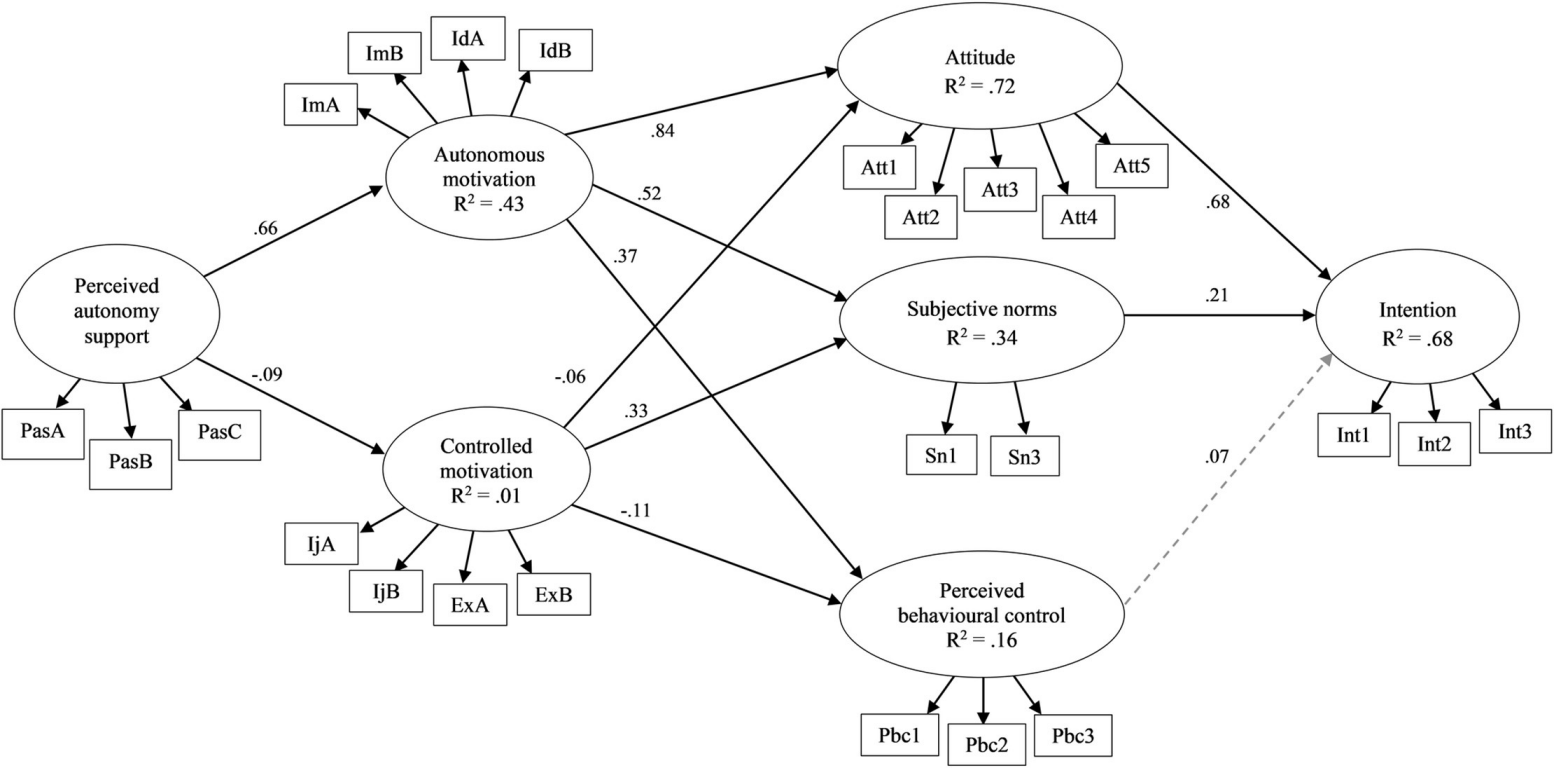
Standardized parameter estimates for the proposed integrated model for the single-group analysis in the full sample in leisure-time context. Estimated covariances between observed variables and between latent variables omitted from the figure for the clarity; arrowed paths with solid lines represent statistically significant paths (*p* < .05) and arrowed paths with dashed lines represent non-significant paths; R^2^ = coefficient of determination.

**Fig 3:**
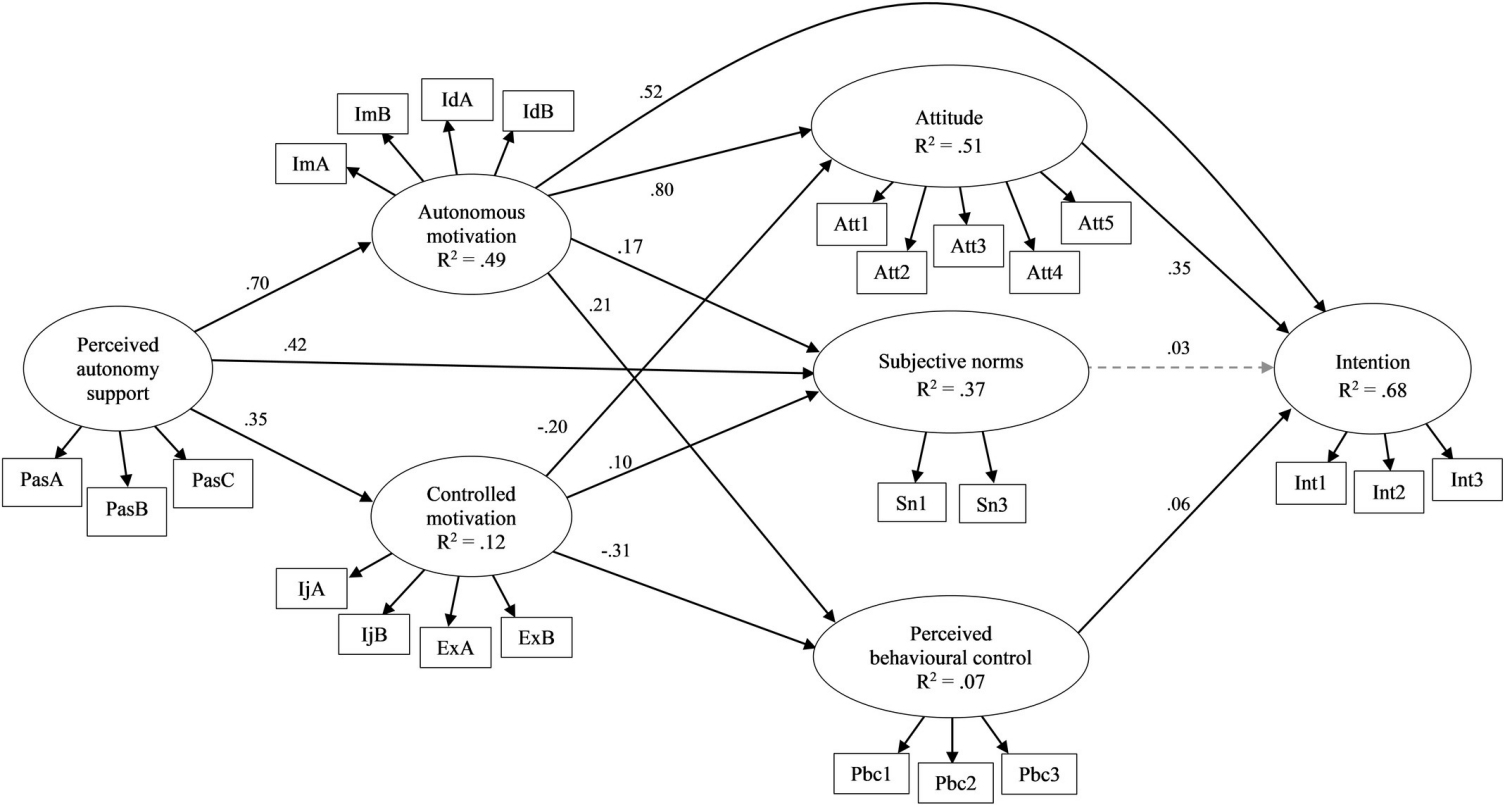
Standardized parameter estimates for the proposed integrated model for the single-group analysis in the full sample in recess context. Estimated covariances between observed variables and between latent variables omitted from the figure for the clarity; arrowed paths with solid lines represent statistically significant paths (*p* < .05) and arrowed paths with dashed lines represent non-significant paths; R^2^ = coefficient of determination.

The hypothesized theoretical model (Fig 1) was tested separately for the leisure-time and recess contexts. The models for the leisure-time (YBχ^2^ = 1685.961, df = 237, *p* < .001, CFI = .868, RMSEA = .085) and recess (YBχ^2^ = 1550.683, df = 237, *p* < .001, CFI = .894, RMSEA = .081) contexts exhibited poor fit with the data. Modification indices suggested inclusion of error covariances among some of the residuals of items from the same scale in both contexts, and direct paths from autonomous motivation to intention and perceived autonomy support by peers to subjective norms were included in the recess context model only. These additional associations are indicated theoretically appropriate and have been added to the model in previous studies [13,32]. Releasing these parameters resulted in well-fitting models for the leisure-time (YBχ^2^ = 610.891, df = 226, *p* < .001, CFI = .965, RMSEA = .045) and recess contexts (YBχ^2^ = 504.278, df = 228, *p* < .001, CFI = .978, RMSEA = .038) (Figs 2 and 3). Overall, the models explained 68% of the variance in intentions in both contexts. The parameter estimates with 95% confidence intervals for model paths in the full sample are presented in Table 1 for both contexts.

**Table 1:** Standardized parameter estimates with 95% confidence intervals from the structural equation model of the integrated model in the leisure-time and recess contexts.

Effect	Leisure-Time			Recess		
	β	CI_.95_		β	CI_.95_	
		LB	UB		LB	UB
PAS→Autonomous motivation	.66***	.59	.71	.70***	.65	.75
PAS→Controlled motivation	-.09*	-.16	-.01^a^	.35***	.29	.41^a^
PAS→Subjective norm	−	−	−	.42***	.31	.53
Autonomous motivation→Attitude	.84***	.80	.88	.80***	.74	.86
Autonomous motivation→Subjective norm	.52***	.44	.59^a^	.17**	.04	.30^a^
Autonomous motivation→PBC	.37***	.29	.46^a^	.21***	.13	.28^a^
Autonomous motivation→Intention	−	−	−	.52***	.42	.62
Controlled motivation→Attitude	-.06*	-.12	-.01	-.20***	-.27	-.12
Controlled motivation→Subjective norm	.33***	.27	.40^a^	.10*	.02	.18^a^
Controlled motivation→PBC	-.11*	-.21	-.01^a^	-.31***	-.39	-.23^a^
Attitude→Intention	.68***	.61	.75^a^	.35***	.27	.44^a^
Subjective norms→Intention	.21***	.14	.28^a^	.03	-.04	.09^a^
PBC→Intention	.07	-.01	.14	.06*	.01	.11
Indirect effects						
PAS→AUT→Attitude→Intention	.37***	.32	.43^a^	.20***	.14	.26^a^
PAS→AUT→SN→Intention	.07***	.05	.10^a^	.00	-.01	.01^a^
PAS→AUT→PBC→Intention	.02	-.00	.04	.01*	.00	.02
PAS→AUT→Intention	−	−	−	.37***	.29	.44
PAS→CON→Attitude→Intention	.00	-.00	.01^a^	-.02**	-.04	-.01^a^
PAS→CON→SN→Intention	-.01	-.01	.01	.00	-.00	.00
PAS→CON→PBC→Intention	.00	.00	.00	-.01*	-.01	.00
PAS→SN→Intention	−	−	−	.01	-.02	.04
AUT→Attitude→Intention	.57***	.50	.64^a^	.28***	.20	.36^a^
AUT→SN→Intention	.11***	.07	.15^a^	.01	-.01	.02^a^
AUT→PBC→Intention	.02	-.00	.05	.01*	.00	.02
CON→Attitude→Intention	-.04*	-.08	-.01	-.07***	-.11	-.03
CON→SN→Intention	.07***	.04	.10^a^	.00	-.00	.01^a^
CON→PBC→Intention	-.01	-.02	.00	-.02*	-.03	-.00
R^2^ AUT	.43	−	−	.49	−	−
R^2^ CON	.01	−	−	.12	−	−
R^2^ Attitude	.72	−	−	.51	−	−
R^2^ Subjective norms	.34	−	−	.37	−	−
R^2^ PBC	.16	−	−	.07	−	−
R^2^ Intention	.68	−	−	.68	−	−

PAS = Perceived autonomy support from peers; AUT = Autonomous motivation; CON = Controlled motivation; PBC = Perceived behavioral control; SN = Subjective norms; CI_95_ = 95% Confidence interval; LB = Lower bound of CI_95_; UB = Upper bound of CI_95_

^a^ = Parameter estimate significantly non-invariant across contexts.

*p < .05

**p < .01

***p < .001.

Although we did not compute an a priori statistical power analysis, a limitation of the current study, we did, however, conduct a post hoc analysis to compute the statistical power of each of the overall models for the leisure-time and recess contexts using the WebPower package in R [50] to ensure that we had sufficient power to detect desired effects. Power was estimated using Satorra and Saris’ [51] formula with the following inputs: the sample size and degrees of freedom from each model, effect size estimated using the formula: YBχ^2^/(n-1), and alpha set at 0.01. Reproduced statistical power was equivalent to 1.00 for both models indicating that we had sufficient statistical power to detect effects in both contexts. Analysis outputs for the post-hoc statistical power analyses are available online: https://osf.io/rtkmh/.

We found support for the hypothesized model paths in the leisure-time context. For ease of expression, all reported paths are positive in direction unless otherwise stated: attitudes and subjective norms predicted intentions; autonomous motivation predicted attitudes, subjective norms, and perceived behavioral control; controlled motivation predicted subjective norms, and negatively predicted attitudes and perceived behavioral control; and perceived autonomy support predicted autonomous motivation, and negatively predicted controlled motivation (Fig 2). Consequently, autonomous motivation, controlled motivation, attitudes, and subjective norms mediated paths from perceived autonomy support to intention (Table 1).

Estimation of the model in the recess context also supported model hypotheses: attitudes and perceived behavioral control predicted intentions; autonomous motivation predicted attitudes, subjective norms, and perceived behavioral control; controlled motivation predicted subjective norms, and negatively predicted attitudes and perceived behavioral control; and perceived autonomy support predicted autonomous and controlled motivation. We included two paths not specified in our hypotheses, but identified as statistically appropriate by modification indices: a path from autonomous motivation to intentions, and a path from perceived autonomy support to subjective norms (Table 1, Fig 3). These additional paths were considered theoretically appropriate as they have been included in previous model tests [13,32], and suggest that the theory of planned behavior variables may inadequately capture effects of motives on intentions, and that subjective norms can reflect beliefs that significant others offer support as well as pressure.

### Tests of invariance

The structural equation models estimated in the full sample were tested for invariance of model parameters across gender, grade, and school groups. Goodness-of-fit statistics for all configural models across the subgroups were all within acceptable limits in the both contexts (detailed information is provided in supporting materials in S5 Table). Compared to the model estimated for in the full sample, modification indices suggested that two additional paths should be included: a path from perceived autonomy support to perceived behavioral control for the model estimated in grade 8 students in the leisure-time context, and a path from controlled motivation to intention in the model estimated in boys and in grade 9 students in the recess context.

As a part of the multigroup structural equation modeling analysis, we found the majority of factor loadings invariant across models, but we also identified some non-invariant factor loadings, these are listed in the notes of Table 2. Constraining paths among latent variables in the SEM to be invariant yielded adequate model fit, supporting invariance across all subgroups in the leisure-time context (Table 2). However, due to a statistically significant ΔSBχ^2^ value and a modification index above 10, the path from autonomous motivation to perceived behavioral control in School C was considered non-invariant and lower than in Schools A and B. Consequently, the path structure was partially invariant across schools and fully invariant across gender and grades in the leisure-time context.

**Table 2:** Results of the multigroup structural equation modeling analysis testing for invariance of model parameters of the proposed integrated model across gender, grade, and school.

	Leisure-time						
	YBχ^2^	df	CFI	RMSEA	ΔSBχ^2^	Δdf	*p*
Invariance test for gender							
Baseline model	972.730	458	.955	.052	-	-	-
λ constrained	1042.786	475	.950	.053	63.063	17	.000
λ partially constrained ^a^	1006.638	473	.953	.052	33.452	15	.004
β constrained	1016.584	484	.953	.051	11.778	11	.381
Invariance test for grades							
Baseline model ^b^	1306.602	687	.948	.057	-	-	-
λ constrained	1363.482	721	.946	.056	58.241	34	.006
λ partially constrained ^c^	1335.370	720	.948	.055	34.469	33	.397
β constrained	1386.226	742	.946	.056	49.587	22	.001
Invariance test for schools							
Baseline model	1208.359	685	.956	.052	-	-	-
λ constrained	1246.713	719	.956	.051	43.061	34	.137
β constrained	1283.737	741	.954	.051	37.142	22	.023
β partially constrained ^d^	1270.062	740	.956	.050	24.750	21	.258
	Recess						
	YBχ^2^	*df*	CFI	RMSEA	ΔSBχ^2^	Δ*df*	*p*
Invariance test for gender							
Baseline model ^e^	845.626	460	.970	.045	-	-	-
λ constrained	870.343	477	.969	.044	27.042	17	.057
β constrained	892.084	490	.969	.044	21.708	13	.060
Invariance test for grades							
Baseline model ^f^	1156.474	694	.964	.049	-	-	-
λ constrained	1239.569	728	.961	.050	75.014	34	.000
λ partially constrained ^g^	1188.634	725	.964	.048	37.952	31	.180
β constrained	1237.172	751	.963	.048	48.305	26	.005
β partially constrained ^h^	1224.452	750	.964	.047	35.952	25	.072
Invariance test for schools							
Baseline model	1106.739	693	.969	.046	-	-	-
λ constrained	1213.416	727	.963	.049	90.801	34	.000
λ partially constrained ^i^	1123.543	724	.970	.044	26.235	31	.710
β constrained	1168.570	750	.968	.045	45.161	26	.011

*Note*. YBχ^2^ = Yuan-Bentler scaled chi-square value of model fit; df = Degrees of freedom for chi-square statistic; CFI = Comparative fit index; RMSEA = Root mean square error of approximation; ΔSBχ^2^ = Incremental change in Satorra-Bentler scaled chi-square value; Δdf = Incremental change in degrees of freedom; *p* = probability of the ΔSBχ^2^

^a^Factor loadings of the intention item 1in the leisure-time context and attitude item 3 in the leisure-time context non-invariant across gender

^b^Additional effect of perceived autonomy support by peers on perceived behavioral control estimated in the grade 8 subsample

^c^Factor loading of the perceived behavioral control item 2 in leisure-time context non-invariant in the grade 9 subsample

^d^Effect of autonomous motivation on perceived behavioral control non-invariant in the School C subsample

^e^Additional effect of controlled motivation on intentions estimated in the boys subsample

^f^Additional effect of controlled motivation on intentions estimated in the grade 9 subsample

^g^Factor loading of the intention item 1 in the recess context non-invariant in the grade 7 subsample, the factor loading of the introjected motivation parceled item A in the recess context non-invariant in the grade 8 subsample, the factor loading of the extrinsic motivation parceled item B in the recess context non-invariant in the grade 9 subsample

^h^Effect of autonomous motivation on intentions and the effect of perceived autonomy support on autonomous motivation non-invariant in the grade 9 subsample

^i^Factor loadings of the extrinsic motivation parceled item A in the leisure-time context and perceived behavioral control item 2 in the recess context non-invariant in the School B subsample, the factor loading of the extrinsic motivation parceled item B in the recess context item non-invariant in the School C subsample.

Turning to the recess context, the path structure was fully invariant across gender and schools. Significant ΔSBχ^2^ and modification indices, suggested two paths should be freely estimated in the model for grade 9 students: the path from perceived autonomy support by peers to autonomous motivation was lower, and the path from autonomous motivation to intention was higher in grade 9 model than the paths in the grade 7 and grade 8 models (Table 2). Consequently, we concluded that the path structure across grades was partially invariant in recess context.

### Models including past behavior

Standardized parameter estimates for the SEM testing paths among constructs in our proposed integrated model for the overall sample controlling for past behavior with 95% confidence intervals are presented in Table 3. The models including past behavior exhibited acceptable fit with the data for models in the leisure-time (YBχ^2^ = 679.142, df = 266, p < .001, CFI = .966, RMSEA = .043) and recess (YBχ^2^ = 562.590, df = 268, p < .001, CFI = .979, RMSEA = .036) contexts. These models explained 77% and 85% of the variance in intentions in leisure-time and recess contexts, respectively. Past behavior was a statistically significant predictor of all model variables with the exception of controlled motivation and perceived behavioral control in the leisure-time context, and subjective norms in the recess context. Most associations remained invariant once past behavior had been included. However, the strength of the paths from perceived autonomy support by peers to autonomous motivation, autonomous motivation to attitudes and attitudes to intentions were attenuated based on 95% confidence intervals in leisure-time context. Specifically, the paths from perceived autonomy support to autonomous motivation, and from autonomous motivation to intentions, in the recess context were attenuated. In addition, the paths from autonomous motivation to subjective norms, and of perceived behavioral control on intention were no longer statistically significant after including past behavior in the recess context model.

**Table 3:** Standardized parameter estimates with 95% confidence intervals from the structural equation models including past behavior for the leisure-time and recess contexts.

Effect	Leisure-Time		Recess	
	β	CI_95_	β	CI_95_
PAS→Autonomous motivation	.46***	.39, .53	.52***	.46, .59
PAS→Controlled motivation	-.07	-.15, .02^a^	.30***	.23, .36 ^a^
PAS→Subjective norm	−	−	.42***	.31, .53
Autonomous motivation→Attitude	.70***	.62, .79	.72***	.63, .82
Autonomous motivation→Subjective norm	.38***	.25, .52	.15	-.02, .31
Autonomous motivation→PBC	.38***	.24, .51	.14*	.03, .25
Autonomous motivation→Intention	−	−	.23***	.13, .32
Controlled motivation→Attitude	-.07**	-.12, -.02	-.18***	-.26, -.10
Controlled motivation→Subjective norm	.34***	.27, .40 ^a^	.10*	.02, .19 ^a^
Controlled motivation→PBC	-.11*	-.21, -.01^a^	-.30***	-.38, -.22 ^a^
Attitude→Intention	.38***	.28, .47	.25***	.18, .31
Subjective norm→Intention	.13***	.07, .19	.06*	.01, .11
PBC→Intention	.09*	.01, .16	.01	-.03, .05
Past behavior→PAS	.38***	.31, .46	.42***	.35, .48
Past behavior→Autonomous motivation	.53***	.47, 60	.44***	.37, .51
Past behavior→Controlled motivation	-.05	-.14, .04 ^a^	.13**	.05, .21 ^a^
Past behavior→Attitude	.20***	.10, .29	.11*	.03, .20
Past behavior→Subjective norm	.20**	.06, .34	.03	-.07, .14
Past behavior→PBC	.00	-.15, .15	.10*	.01, .19
Past behavior→Intention	.46***	.37, .55	.55***	.47, .62
Indirect effect				
PAS→AUT→Attitude→Intention	.12***	.08, .16	.09***	.06, .13
PAS→AUT→SN→Intention	.02**	.01, .05	.00	-.00, .01
PAS→AUT→PBC→Intention	.02*	.00, .03	.00	-.00, .00
PAS→AUT→Intention	−	−	.12***	.07, .17
PAS→CON→Attitude→Intention	.00	-.00, .00 ^a^	-.01**	-.02, -.01 ^a^
PAS→CON→SN→Intention	-.00	-.01, .00	.00	.00, .00
PAS→CON→PBC→Intention	.00	-.00, .00	-.00	-.00, .00
PAS→SN→Intention	−	−	.02*	.00, .05
AUT→Attitude→Intention	.27***	.19, .34	.18***	.12, .24
AUT→SN→Intention	.05***	.02, .08	.01	-.00, .02
AUT→PBC→Intention	.03*	.00, .06	.00	-.00, .01
CON→Attitude→Intention	-.03*	-.05, -.01	-.05**	-.07, -.02
CON→SN→Intention	.04***	.02, .06 ^a^	.01	-.00, .01 ^a^
CON→PBC→Intention	-.01	-.02, .00	-.00	-.02, .01
R^2^ Perceived autonomy support	.15		.17	−
R^2^ Autonomous motivation	.68		.65	−
R^2^ Controlled motivation	.01		.14	−
R^2^ Attitude	.74		.52	−
R^2^ Subjective Norm	.37		.37	−
R^2^ Perceived behavioral control	.16		.17	−
R^2^ Intention	.77		.85	−

*Note*. PAS = Perceived autonomy support by peer; AUT = Autonomous motivation; CON = Controlled motivation; SN = Subjective Norm; PBC = Perceived Behavioral Control; CI_.95_ = 95% Confidence interval

^a^Estimate significantly different across contexts.

*p < .05

**p < .01

***p < .001.

**Fig 4:**
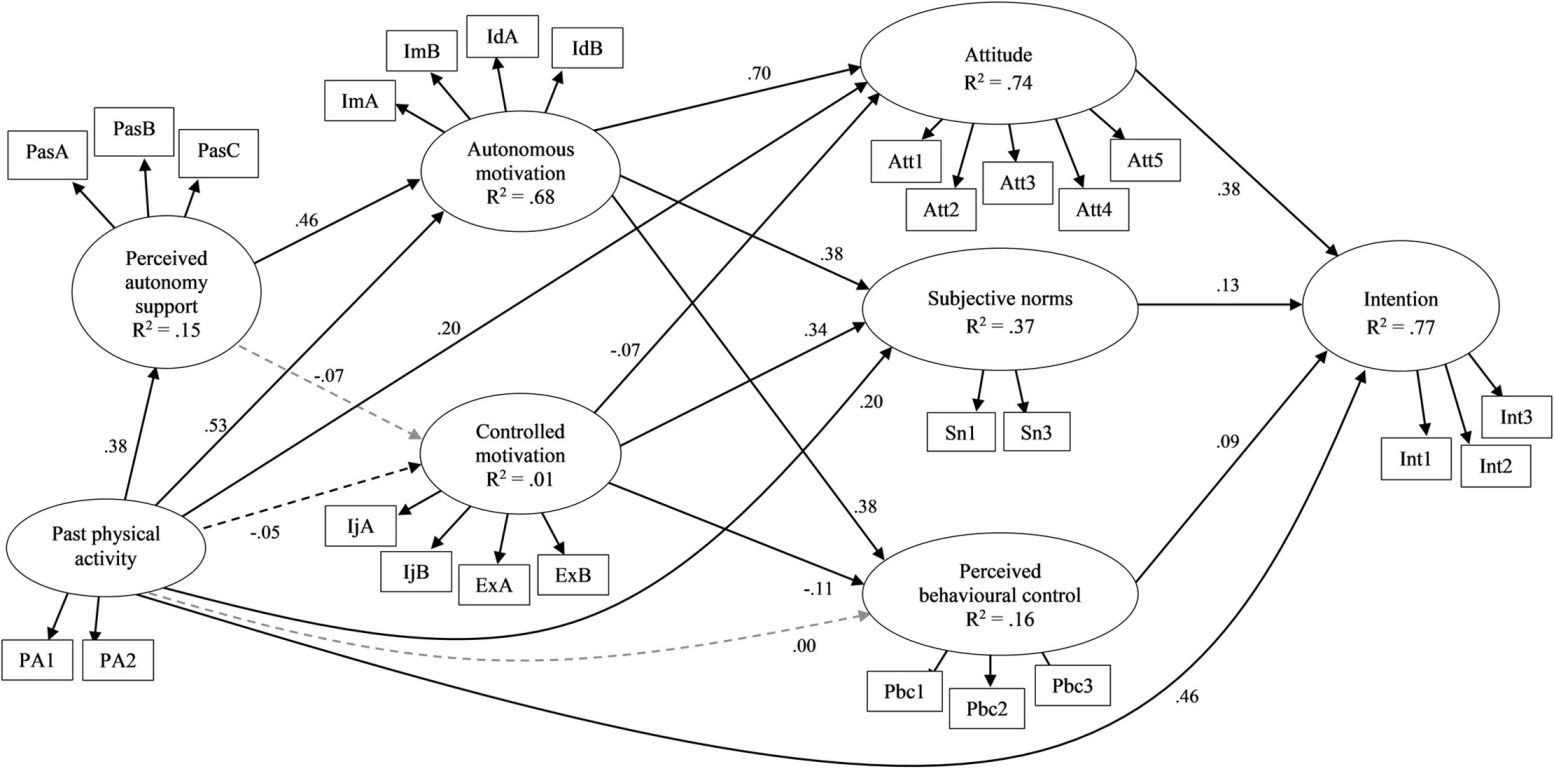
Standardized parameter estimates for the proposed integrated model for the single-group analysis in the full sample with past behavior in leisure-time context. Estimated covariances between observed variables and between latent variables omitted from the figure for the clarity; arrowed paths with solid lines represent statistically significant paths (*p* < .05) and arrowed paths with dashed lines represent non-significant paths; R^2^ = coefficient of determination.

Focusing on model including past behavior estimated in the leisure-time context (Fig 4), attitudes, subjective norms and perceived behavioral control were significant predictors of intentions as hypothesized. Autonomous motivation was a significant predictor of attitudes, subjective norms, and perceived behavioral control, while controlled motivation was a significant predictor of subjective norms, and significant, negative predictor of attitudes and perceived behavioral control. Perceived autonomy support positively predicted autonomous motivation, but did not predict controlled motivation. Importantly, perceived autonomy support had significant indirect paths to intention through autonomous motivation, attitudes, subjective norms, and perceived behavioral control.

**Fig 5:**
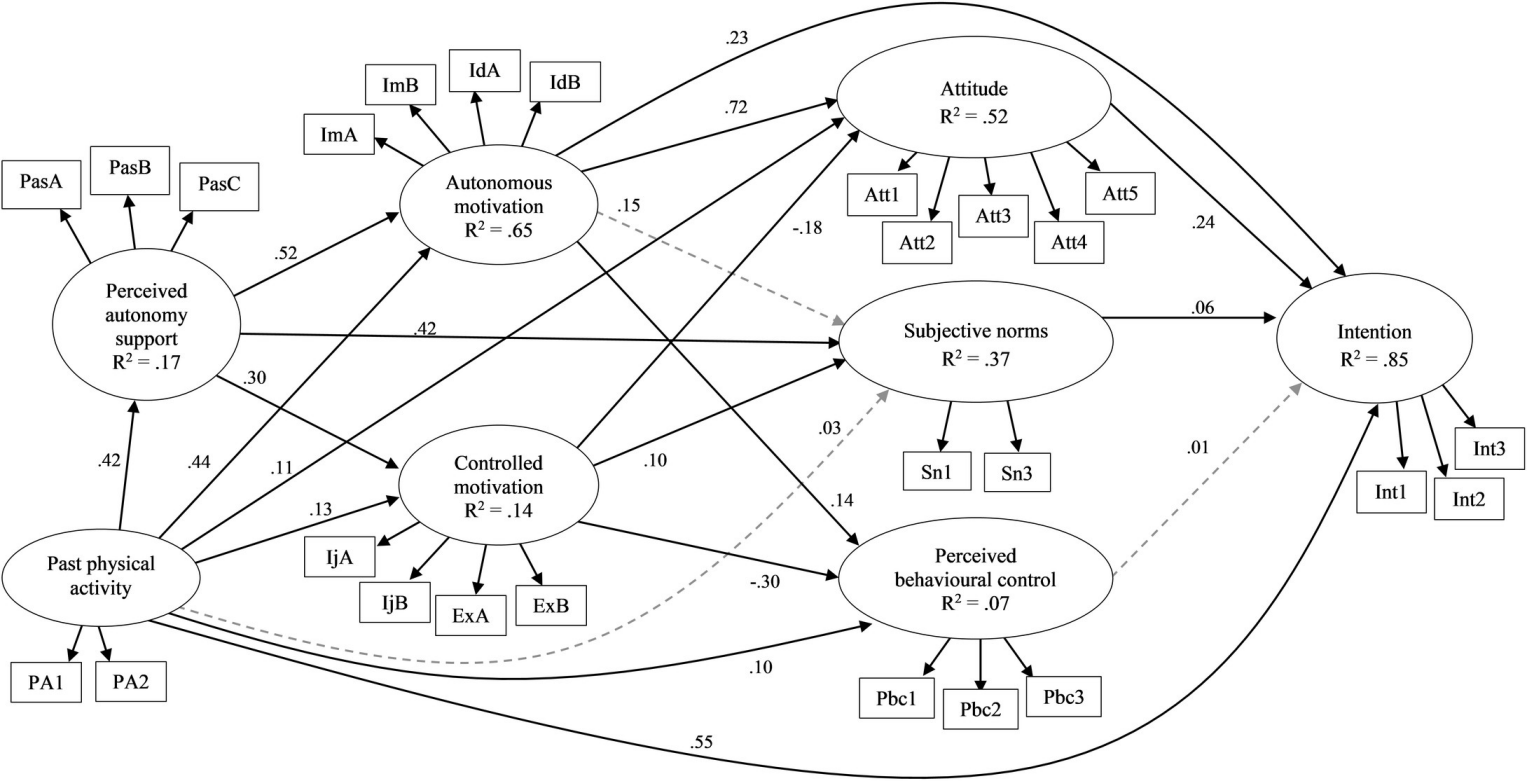
Standardized parameter estimates for the proposed integrated model for the single-group analysis in the full sample with past behavior in recess context. Estimated covariances between observed variables and between latent variables omitted from the figure for the clarity; arrowed paths with solid lines represent statistically significant paths (*p* < .05) and arrowed paths with dashed lines represent non-significant paths; R^2^ = coefficient of determination.

Turning to the model including past behavior estimated in the recess context (Fig 5), attitudes and subjective norms were significant predictors of intentions. Autonomous motivation predicted attitudes and perceived behavioral control, but not subjective norms. Controlled motivation was a predictor of subjective norms, and a negative predictor of attitudes and perceived behavioral control. Perceived autonomy support predicted autonomous and controlled motivation. We also retained the two paths not specified in our hypotheses, but identified a previous stage of the analysis: the effect of autonomous motivation on intentions, and the effect of perceived autonomy support on subjective norms. Perceived autonomy support had an indirect path to attitudes and perceived behavioral control through both autonomous and controlled motivation, and to subjective norms through controlled motivation. The indirect path from perceived autonomy support to intentions was mediated by both autonomous and controlled motivation and attitudes, controlled motivation and subjective norms, and through autonomous motivation and subjective norms alone.

We also compared model paths for the model estimated in the full sample across contexts using 95% confidence intervals of the parameter estimates after controlling for past behavior (Table 3). Most of the paths were no different across the two contexts. Only the path from controlled motivation to subjective norms was larger in the leisure-time context, and the paths from controlled motivation to perceived behavioral control, and from perceived autonomy support to controlled motivation were larger in recess context. In addition, the path from past physical activity to controlled motivation was larger in the recess context.

## Discussion

We aimed to identify the social cognition and motivational correlates of school students’ intentions to participate in leisure-time and school recess physical activity using an integrated theoretical model based on self-determination theory [23] and the theory of planned behavior [21]. We tested model tenets in a sample of lower secondary school students from three schools in Finland. We also tested whether model parameters differ across the leisure-time and school recess contexts. Finally, we tested the invariance of model constructs across context, gender, grade, and school. We expected few variations given that model paths are assumed to be generalizable across population and context. Our study is unique as it examines the key theory-based correlates of children’s physical activity intentions in two important contexts. School recess is a particularly pertinent context given the paucity of research on the antecedents of physical activity intentions in this context and its potential to inform interventions to promote physical activity participation. While there has been general advocacy for the identification of context-specific correlates of physical activity [20], a key assumption of the theoretical models underpinning the research is that their effects will generalize across contexts and populations [21,22]. This presents a somewhat opposing view to the proposal that correlates of physical activity should be context-specific. One possibility is that the constructs themselves do not vary across contexts, rather the relatively contribution of each to the prediction of physical activity intentions varies. We therefore compared the proposed relations among constructs from the proposed integrated model effects across contexts.

Results indicated that perceived autonomy support from peers was consistently related to students’ physical intentions across contexts. This association was indirect, and mediated by autonomous motivation, controlled motivation, attitude and subjective norms in leisure-time, and by autonomous motivation, controlled motivation, attitude and perceived behavioral control at recess. However, the theory of planned behavior variables did not fully mediate the path from autonomous motivation to intention at recess, and autonomous motivation directly predicted intention. Inclusion of past behavior attenuated model paths, and likely reflects the fact that students may have formed similar motives and beliefs previously, and that those constructs were related to intentions to engage in physical activity in the past [52,53]. The relationships, therefore, may be indicative of habitual or routine decision making consistent with dual process models of action [53–55].

Overall, our results highlight the importance of autonomous motivation and attitude as correlates of intentions to participate in physical activity in both leisure-time and school recess contexts. Furthermore, attitudes consistently mediated the effect of autonomous motivation on intention in both contexts. These findings are consistent with previous research identifying autonomous forms of motivation as an important correlate of intentions to participate physical activity in future [27,41], and extend them to physical activity in recess. Consistent with theory, these findings suggest that a potential mechanism by which motives reflecting self-endorsed reasons for being active translate into intentions to seek out future physical activity opportunities is through the belief systems that underpin intention formation. In other words, school children who are autonomously motivated to perform physical activity in leisure-time and recess likely align their beliefs about the activity with their motives, and form intentions to perform activity in the future consistent with those beliefs [13,56].

In contrast, subjective norms and perceived behavioral control had relatively small or trivial association with intentions, in both contexts. Previous research has typically found relatively modest effects for subjective norms on intentions to engage in physical activity [57]. This is despite research that has indicated an important influence of normative factors on health behaviors like physical activity [58]. A likely issue is related to measurement, given that subjective norms tend to be measured as social pressure, more akin to controlled motivation, than social support. The modest paths from controlled motivation to subjective norm in this current study support this presumption. Research has suggested that operationalization of normative influence as descriptive norms [59], that is, beliefs reflecting identification with others who perform the behavior, and social support [58], reflecting positive normative support may be more relevant as social influences, and is a factor that warrants greater exploration.

The small, non-significant relationship between perceived behavioral control and intention diverges from previous results [24,30]. Control, and related constructs such as self-efficacy, have been consistently identified as important correlates of intention and behavior [60]. The modest paths were also not attributable to past behavior. One possible reason may be the strength of the correlations between attitudes and perceived behavioral control. This suggests considerable overlap in these constructs, which has been identified previously [61]. So it is possible that the relatively modest impact may have been due to measurement issues, and the current measure may have been insufficient in capturing control-related beliefs. Perhaps adopting more specific forms of self-efficacy beliefs, such as beliefs about overcoming barriers may have led to stronger associations, as indicated in previous research [61]. This is a speculative account and warrants future research. Of course, we should not rule out the possibility that control-related beliefs are not that relevant in these contexts, and the low zero-order correlation between perceived behavioral control and intention seems to support this. This may especially be the case in recess, where the notion of volitional control may actually be less relevant than personal beliefs, such as attitudes. Perhaps there is no problems of capacity and volition in these contexts, rather it is personal beliefs about outcomes that are most relevant.

Although there have been numerous studies examining the effects of social cognition and motivational factors on physical activity intentions in leisure-time [for review see 32], and in PE lessons [13], there has been a relative dearth of research on recess physical activity. Our research, therefore, advances knowledge of the antecedents of physical activity given that previous research examining physical activity in recess has mainly focused on structural and environmental factors [62]. Consistent with the broad assumption of generalizability of the component theories of the integrated model [63], we proposed that relationships would hold regardless of population characteristics and context. Our between-group invariance analyses across gender, grade, and school, as well as within group comparisons across contexts, largely supported this premise. However, we did identify some variations, which may have implications for understanding antecedents of physical activity intentions in secondary school students. The most notable differences in model paths across contexts were the additional paths in the recess context: the path from perceived autonomy support to subjective norms and path from autonomous motivation to intention. In addition, the paths from controlled motivation to subjective norms and perceived behavioral control, the effect of perceived autonomy support on controlled motivation, were statistically different between contexts after controlling for past behavior.

It seems that controlled motivation had pervasive negative paths to attitudes and perceived behavioral control. This resulted negative indirect paths from controlled motivation to intentions through attitudes in both contexts, which contrasted with the positive indirect path from controlled motivation to intentions through subjective norms in leisure-time. This seems to indicate the importance of reducing controlled forms of motivation in the school context, given the potential for controlled reasons to undermine physical activity intentions. This is consistent with research suggesting that interventions to promote physical activity participation should not focus on promoting autonomous motivation alone, but should also focus on reducing controlled motivation (e.g., through reducing use of controlling language and rewards and punishments by teachers; [64,65]). In contrast, it seems controlled motivation may be effective in intention formation for physical activity through subjective norms in leisure-time. To speculate, is possible that leisure-time physical activity may, for some young people, may be motivated by externally referenced reasons, for example to improve physical appearance, to please parents, or to win in sports. Nevertheless, this may be for a small proportion of the population, given that indirect paths from autonomous motivation through attitudes were much larger in both samples.

The positive association between perceived autonomy support and controlled motivation in the recess period was also unexpected, and seems counter intuitive given the purpose of autonomy support is to promote autonomous rather than controlled motivation. This path also contrasts with previous research that has tended to demonstrate weak or null correlations between these constructs [41]. A possible explanation may lie in students’ interpretation of support from their peers. Some students, for example, may interpret some of the behaviors that are ostensibly supportive, such as listening and positive feedback, as potentially controlling. This is consistent with the notion that individuals can interpret the behaviors and body language of others in different ways. There is also the proposal that there are individual differences in causality orientations that is whether individuals have a general tendency to interpret ambiguous behaviors or situations as either autonomy supportive or controlling [66,67]. It is possible that individuals with controlled causality orientations may interpret certain behaviors as controlled even though those with neutral or autonomous causality orientations interpret them otherwise [68]. However, in the absence of any data on causality orientations to corroborate these hypotheses, it remains speculative.

Perceived autonomy support by peers had the most consistent positive indirect effects on physical activity intentions through autonomous forms of motivation across contexts. This highlights the importance of support from peers as a correlate of intentions to participate in future physical activity in both contexts, and is consistent with previous research [69]. This is also consistent with the values that adolescents attach to the support and endorsement of their friends and associates when engaging in behaviors [70]. This highlights a potential avenue for intervention: by providing opportunities for peers to mutually support their motivation to participate in physical activity both within and outside of school.

### Strengths, limitations, and directions for future research

The present study had a number of strengths including the adoption of an integrated theoretical model aimed at identifying the key motivational antecedents of physical activity participation, a focus on two contexts in which lower secondary school students have the opportunity to participate in physical activity, collection of data on a large sample of secondary school students, and adoption of a rigorous analytic approach which allowed for between group comparisons.

These strengths notwithstanding, it is also important to note some aspects of the study that might limit interpretation and generalizability. First, the study design is cross-sectional and directional inferences of a causal nature for the proposed paths are based on theory, not the data. Future research should consider cross-lagged panel, experimental, and intervention designs to evaluate construct change and causation in physical activity intentions.

Second, it is important to note that we did not measure perceived autonomy support from other sources such as teachers or parents, sources of autonomy support expected to have pervasive associations with students’ physical activity participation recess and leisure-time. In fact, there is evidence that students’ perceptions of their PE teachers’ support for autonomy is also related to their physical activity participation in leisure-time [32,71]. So, an important direction for future research would be to evaluate students’ perceptions of all potential sources of autonomy support on physical activity participation in both contexts. It is also important to note that perceived autonomy support does not necessarily equate to actual autonomy support, so an extension of the present study may be to compliment measures of perceived autonomy support with externally verified measures of actual autonomy support, for example through use of an observation tool.

Third, we focused year group and, therefore, used grade as a moderator of model effects in the current analysis. However, such an analysis does not account for biological maturation compared to age, and maturation is likely to be a potential influence on physical activity levels and its determinants, and we did not collect data on maturity in the current study, so we were not able to detect differences in model effects due to maturity, a potential limitation of the current analysis. However, importance of social influence from adolescents of the same age on participants’ physical activity behavior and its determinants should not be discounted. Students spend most of their time with their classmates in the same age cohort at school, and also tend co-act in physical activity with peers of similar ages outside of school. We therefore, we hypothesize that the effect of social context is likely to have had a pervasive influence on their physical activity and its determinants. As a consequence, examining differences between grades still has meaning in terms of understanding differences in physical activity participation and its determinants in the current research. Nevertheless, future studies should consider collecting data in biological maturity alongside age, and test each as a moderator of model effects.

Finally, we focused solely on physical activity intentions rather than actual behavior. Although the theory of planned behavior, a component theory of the integrated model tested here, indicate that intentions are a direct antecedent of behavior, research suggests that the relationship, while usually statistically significant, varies considerably and is seldom perfect [72]. Certainly previous research indicates that effects of school students’ intentions on leisure-time physical activity participation is medium in size [13]. This intention-behavior gap suggests that intentions alone may not result in behavioral engagement. Future research should not only consider testing the intention-behavior relationship in school recess, and compare it to leisure-time effects, there should also be considerations of the factors that might moderate that relationship, such as intention stability and planning capacity.

## Conclusions

The current study aimed to predict secondary school students’ intentions to participate in physical activity in school recess and leisure-time contexts using an the integrated motivational and social cognition model deriving its hypotheses from multiple theories. We proposed that adolescents’ physical activity intentions in both contexts would be a function of their perceived autonomy support from peers, and their autonomous forms of motivation and social cognition factors with respect to performing physical activity in each context in future. Predictions were tested large sample of Finnish adolescents attending lower secondary school using a correlational design. Results supported relations between the motivational and social cognition constructs and physical activity intentions consistent with the model predictions. Specifically, autonomous motivation and attitude toward physical activity fully mediated effects of perceived autonomy support from peers on students’ physical intentions in recess and leisure-time contexts. However, the theory of planned behavior constructs did not fully mediate effects of autonomous motivation on intentions in the recess context, as autonomous motivation predicted intention directly. Including past behavior did not substantially diminish model effects. In addition, model effects were consistent across school, grade, and gender. Our study contributed to the evidence base of the correlates of secondary school students’ intentions to participate in physical activity in leisure-time and school recess contexts. These data may assist in the identification of targets for school-based interventions to promote physical activity. Future research could include follow-up measures of physical activity, and intervention designs, where students are encouraged to support their peers to be physically active.

## Supporting information

S1 Table Scale items for measures of constructs of the integrated model for leisure-time physical activity. (DOCX)

S2 Table Scale items for measures of constructs of the integrated model for recess physical activity. (DOCX)

S3 Table Descriptive statistics, composite reliabilities, average variance extracted, and standardized factor correlations for latent variables from the structural equation modeling analysis of the integrated model in the leisure time and recess contexts. (DOCX)

S4 Table Results of the multigroup confirmatory factor analysis testing invariance of model parameters across gender, grade and school in the leisure time and recess contexts. (DOCX)

S5 Table Goodness-of-fit statistics for single-group structural equation models for full sample, and gender, grade and school sub-samples in the leisure time and recess contexts. (DOCX)

S1 Appendix Parceling process applied for autonomous motivation, controlled motivation, and perceived autonomy support by peers constructs. (DOCX)
