# Supplementary material for: Predicting school students’ physical activity intentions in leisure-time and school recess contexts: Testing an integrated model based on self-determination theory and theory of planned behavior
Source: PLoS One. 2021 Mar 26;16(3):e0249019. doi: 10.1371/journal.pone.0249019 (PMC7997014; doi:10.1371/journal.pone.0249019)
Supplement: S1 Table — (DOCX) [file pone.0249019.s001.docx]

**S1 Table**. **Scale Items for Measures of Constructs of the Integrated Model for Leisure-Time Physical Activity**

| Variable | Item | Scale |
| --- | --- | --- |
| Intention | I intend to do physical activities, for at least 20 minutes, during my free time, over the next 4 weeks with the following regularity. (Int1_LT)  I intend to do physical activities, for at least 20 minutes, 3 days per week during my free time, over the next 4 weeks. (Int2_LT)  I intend to do physical activities, for at least 20 minutes, 3 days per week during my free time, over the next 4 weeks. (Int3_LT) | 1 = “not at all”, 7 = “every day”  1 = “unlikely”, 7 = ”very likely”.  1 = “definitely not”, 6 = “definitely” |
| Attitude | My doing physical activities, for at least 20 minutes, 3 days per week over the next 4 weeks during my free time is… | 1= “unenjoyable”, 7 = “enjoyable” (Att1_LT)  1= “bad”, 7 = “good” (Att2_LT)  1 = “useless”, 7 = “useful” (Att3_LT)  1 = “boring”, 7 = “interesting” (Att4_LT)  1 = “harmful”, 7 = “beneficial” (Att5_LT) |
| Subjective norm | Most people who are important to me would want me to do physical activities for at least 20 minutes, 3 days per week during my free time over the next 4 weeks. (Sn1_LT)  Most people I know would approve of me doing physical activities for at least 20 minutes, 3 days per week during my free time over the next 4 weeks. (Sn2_LT)  Most people close to me expect me to do physical activities for at least 20 minutes, 3 days per week during my free time over the next 4 weeks. (Sn3_LT) | 1 = “strongly disagree”, 7 = “strongly agree”  1 = “strongly disagree”, 7 = “strongly agree”  1 = “strongly disagree”, 7 = “strongly agree” |
| Perceived behavioral control | How much control do you have over doing physical activities for at least 20 minutes, 3 days per week during your free time in the next 4 weeks? (Pbc1_LT)  If I wanted to I could do physical activities for at least 20 minutes, 3 days per week during my free time over the next 4 weeks. (Pbc2_LT)  I feel in complete control over whether I will do physical activities for at least 20 minutes, 3 days per week during my free time over the next 4 weeks. (Pbc3_LT) | 1 = “I have no control”, 7 = “I have complete control”  1 = “strongly disagree, 7 = “strongly agree”  1 = “I have no control”, 7 = “I have complete control” |
| Autonomous motivation | I do physical activities during my freetime…  … Because I value the benefits of physical activity (Id1_LT)  … Because it is fun. (Im1_LT)  … Because it’s important to me to do physical activities regularly. (Id2_LT)  … Because I enjoy my physical activity sessions. (Im2_LT)  … Because I think it’s important to make the effort to do physical activities regularly. (Id3_LT)  … Because I find physical activities as a pleasurable activity. (Im3_LT)  … Because I get restless if I don’t do physical activities regularly. (Id4_LT)  … Because I get pleasure and satisfaction from participating in physical activities. (Im4_LT) | 1 = “not at all true”, 5= “very true”  1 = “not at all true”, 5= “very true”  1 = “not at all true”, 5= “very true”  1 = “not at all true”, 5= “very true”  1 = “not at all true”, 5 = “very true”  1 = “not at all true”, 5 = “very true”  1 = “not at all true”, 5 = “very true”  1 = “not at all true”, 5 = “very true” |
| Controlled motivation | I do physical activities during my freetime…  … Because other people say I should. (Ex1_LT)  … Because feel guilty when I don’t do physical activities. (Ij1_LT)  … Because my people important to me (parents, family etc.) say I should. (Ex2_LT)  … Because I feel ashamed when I miss a physical activity session (Ij2_LT)  … Because other people will not be pleased with me if I don’t do physical activities. (Ex3_LT)  … Because I feel like a failure when I haven’t been physically active in a while. (Ij3_LT)  … Because I feel under pressure from my friends/family to do physical activities. (Ex4_LT)  … Because I will feel bad about myself when I don’t do physical activities. (Ij4_LT) | 1 = “not at all true”, 5= “very true”  1 = “not at all true”, 5= “very true”  1 = “not at all true”, 5= “very true”  1 = “not at all true”, 5= “very true”  1 = “not at all true”, 5 = “very true”  1 = “not at all true”, 5 = “very true”  1 = “not at all true”, 5 = “very true”  1 = “not at all true”, 5 = “very true” |
| Perceived autonomy support by peers | My friends encourage me to do physical activities in my free time. (Pas1_LT)  My friends display confidence in my ability to physical activities in my free time. (Pas2_LT)  My friends help me to do physical activities in my free time. (Pas3_LT)  My friends support me when I do physical activities in my free time. (Pas4_LT)  My friends provide me with positive feedback when I do physical activities in my free time. (Pas5_LT)  I am able to talk with my friends about physical activities I do in my free time. (Pas6_LT)  My friends care about the physical activities I do in my free time. (Pas7_LT) | 1 = “strongly disagree”, 7 = “strongly agree”  1 = “strongly disagree”, 7 = “strongly agree”  1 = “strongly disagree”, 7 = “strongly agree”  1 = “strongly disagree”, 7 = “strongly agree”  1 = “strongly disagree”, 7 = “strongly agree”  1 = “strongly disagree”, 7 = “strongly agree”  1 = “strongly disagree”, 7 = “strongly agree” |
| Physical activity | Over a typical or usual week, how often are you physically active and sweat and get out of breath outside school hours? (Pa1_LT)  Over a typical or usual week, how many hours are you physically active and sweat and get out of breath outside school hours? (Pa2_LT) | 1 = “daily”, 7 = “never”  1 = “not at all”, 7 = “7 hours or more” |
