# Supplementary material for: Predicting school students’ physical activity intentions in leisure-time and school recess contexts: Testing an integrated model based on self-determination theory and theory of planned behavior
Source: PLoS One. 2021 Mar 26;16(3):e0249019. doi: 10.1371/journal.pone.0249019 (PMC7997014; doi:10.1371/journal.pone.0249019)
Supplement: S2 Table — (DOCX) [file pone.0249019.s002.docx]

**S2 Table. Scale Items for Measures Constructs of the Integrated Model for Recess Physical Activity**

| Variable | Item | Scale |
| --- | --- | --- |
| Intention | I intend to do physical activities at recess over the next week with the following regularity. (Int1_Re)  I intend to do physical activities at least at one recess per day over the next week. (Int2_Re)  I intend to do physical activities at least at one recess per day over the next week. (Int3_Re) | 1 = “not at all”, 7 = ”every day”.  1 = “definitely not”, 7 = “definitely”  1 = “very unlikely”, 6 = “very likely” |
| Attitude | Doing physical activities at least at one recess per day over the next week is… | 1= “unenjoyable”, 7 = “enjoyable” (Att1_Re)  1= “bad”, 7 = “good” (Att2_Re)  1 = “useless”, 7 = “useful” (Att3_Re)  1 = “boring”, 7 = “interesting” (Att4_Re)  1 = “harmful”, 7 = “beneficial” (Att5_Re) |
| Subjective norm | Most people who are important to me would want me to do physical activities at least at one recess per day over the next week. (Sn1_Re)  Most people I know would approve of me do physical activities at least at one recess per day over the next week. (Sn2_Re)  Most people close to me expect me to do physical activities at least at one recess per day over the next week. (Sn3_Re) | 1 = “strongly disagree”, 7 = “strongly agree”  1 = “strongly disagree”, 7 = “strongly agree”  1 = “strongly disagree”, 7 = “strongly agree” |
| Perceived behavioral control | How much control do you have over doing physical activities at recess over the next week? (Pbc1_Re)  If I wanted to, I could do physical activities at least at one recess per day over the next week. (Pbc2_Re)  I feel in complete control over whether I will do physical activities at least at one recess per day over the next week. (Pbc3_Re) | 1 = “I have no control”, 7 = “I have complete control”  1 = “strongly disagree, 7 = “strongly agree”  1 = “strongly disagree, 7 = “strongly agree” |
| Autonomous motivation | I do physical activities during recess…  … Because I value the benefits of doing physical activities at recess. (Id1_Re)  … Because it is fun. (Im1_Re)  … Because it is important to me to do physical activities regularly at recess. (Id2_Re)  … Because I enjoy my physical activity sessions at recess. (Im2_Re)  … Because I think, it is important to make the effort to do physical activities at recess. (Id3_Re)  … Because I find doing physical activities at recess as a pleasurable activity. (Im3_Re)  … Because I get restless if I don’t do physical activities regularly at recess. (Id4_Re)  … Because I get pleasure and satisfaction from doing physical activities at recess. (Im4_Re) | 1 = “not at all true”, 5= “very true”  1 = “not at all true”, 5= “very true”  1 = “not at all true”, 5= “very true”  1 = “not at all true”, 5= “very true”  1 = “not at all true”, 5 = “very true”  1 = “not at all true”, 5 = “very true”  1 = “not at all true”, 5 = “very true”  1 = “not at all true”, 5 = “very true” |
| Controlled motivation | I do physical activities during recess…  … Because other people say I should. (Ex1_Re)  … Because feel guilty when I don’t do physical activities at recess. (Ij1_Re)  … Because my people important to me demand I should. (Ex2_Re)  … Because I feel ashamed when I miss a change for a physical activity at recess. (Ij2_Re)  … Because other people will not be pleased with me if I don’t do physical activities at recess. (Ex3_Re)  … Because I feel like a failure when I haven’t been physically active in a while at recess. (Ij3_Re)  … Because I feel under pressure from my friends and teachers to do physical activities at recess. (Ex4_Re)  … Because I will feel bad about myself when I don’t do physical activities at recess. (Ij4_Re) | 1 = “not at all true”, 5= “very true”  1 = “not at all true”, 5= “very true”  1 = “not at all true”, 5= “very true”  1 = “not at all true”, 5= “very true”  1 = “not at all true”, 5 = “very true”  1 = “not at all true”, 5 = “very true”  1 = “not at all true”, 5 = “very true”  1 = “not at all true”, 5 = “very true” |
| Perceived autonomy support by peers | My friends encourage me to do physical activities at recess. (Pas1_Re)  My friends display confidence in my ability to do physical activities at recess. (Pas2_Re)  My friends help me to do physical activities at recess. (Pas3_Re)  My friends support me to participate in physical activities at recess. (Pas4_Re)  My friends provide me with positive feedback when I participate in physical activities at recess. (Pas5_Re)  I am able to talk about doing physical activities at recess. (Pas6_Re)  My friends are interested in me participating in physical activities at recess. (Pas7_Re) | 1 = “strongly disagree, 7 = “strongly agree”  1 = “strongly disagree, 7 = “strongly agree”  1 = “strongly disagree, 7 = “strongly agree”  1 = “strongly disagree, 7 = “strongly agree”  1 = “strongly disagree, 7 = “strongly agree”  1 = “strongly disagree, 7 = “strongly agree”  1 = “strongly disagree, 7 = “strongly agree” |
| Physical activity | During the last three weeks: On how many lunch breaks you have been doing physical activities at least half of the break time on average? (Pa1_Re)  During the last three weeks: On how many regular 10 or 15 minutes recess you have been doing physical activities at least half of the recess time on average? (Pa2_Re) | 1 = “I have not been active at lunch breaks”,  6 = “I have been active at all lunch breaks”  1 = “I have not been active at recess”,  6 = “I have been active at all regular breaks” |
