# Supplementary material for: Predicting school students’ physical activity intentions in leisure-time and school recess contexts: Testing an integrated model based on self-determination theory and theory of planned behavior
Source: PLoS One. 2021 Mar 26;16(3):e0249019. doi: 10.1371/journal.pone.0249019 (PMC7997014; doi:10.1371/journal.pone.0249019)
Supplement: S3 Table — (DOCX) [file pone.0249019.s003.docx]

**S4 Table. Descriptive Statistics Composite Reliabilities, Average Variance Extracted, and Standardized Factor Correlations for Latent Variables from the Structural Equation Modeling Analysis of the Integrated Model in the Leisure Time and Recess Contexts**

|  | M | SD | CR | 1 | 2 | 3 | 4 | 5 | 6 | 7 | 8 | 9 | 10 | 11 | 12 | 13 | 14 | 15 | 16 |
| --- | --- | --- | --- | --- | --- | --- | --- | --- | --- | --- | --- | --- | --- | --- | --- | --- | --- | --- | --- |
| 1. Past PA LT  Frequency  Time used | 5.42  4.24 | 1.14  1.29 | .79 | .65 |  |  |  |  |  |  |  |  |  |  |  |  |  |  |  |
| 2. Past PA Re  Lunch Break  Regular Break | 2.19  2.20  2.17 | 1.41  1.55  1.43 | .88 | .08^*^ | .79 |  |  |  |  |  |  |  |  |  |  |  |  |  |  |
| 3. PAS LT | 4.37 | 1.28 | .89 | .37^***^ | .03 | .74 |  |  |  |  |  |  |  |  |  |  |  |  |  |
| 4. AUT LT | 3.49 | .87 | .86 | .69^***^ | .11^**^ | .57^***^ | .61 |  |  |  |  |  |  |  |  |  |  |  |  |
| 5. CON LT | 1.73 | .68 | .80 | -.08 | -.06 | -.04 | -.11^**^ | .52 |  |  |  |  |  |  |  |  |  |  |  |
| 6. ATT LT | 5.97 | 1.04 | .87 | .67^***^ | .08^*^ | .51^***^ | .80^***^ | -.12^**^ | .58 |  |  |  |  |  |  |  |  |  |  |
| 7. SN LT ^a^ | 4.59 | 1.46 | .79 | .45^***^ | -.01 | .40^***^ | .44^***^ | .29^***^ | .41^***^ | .65 |  |  |  |  |  |  |  |  |  |
| 8. PBC LT | 6.12 | 1.05 | .85 | .23^***^ | -.01 | .27^***^ | .30^***^ | -.22^***^ | .39^***^ | .28^***^ | .66 |  |  |  |  |  |  |  |  |
| 9. INT LT  Items 1 & 2  Item 3 | 5.37  4.73 | 1.50  1.34 | .92 | .82^***^ | .11^**^ | .37^***^ | .66^***^ | -.05 | .78^***^ | .52^***^ | .34^***^ | .79 |  |  |  |  |  |  |  |
| 10. PAS Re | 2.77 | 1.43 | .93 | .02 | .41^***^ | .42^***^ | .16^***^ | .01 | .12^**^ | .10^**^ | .02 | .07^**^ | .82 |  |  |  |  |  |  |
| 11. AUT Re | 1.79 | .87 | .91 | .10^**^ | .65^***^ | .21^***^ | .21^***^ | .03 | .15^***^ | .08^**^ | -.02 | .14^***^ | .69^***^ | .73 |  |  |  |  |  |
| 12. CON Re | 1.21 | .45 | .91 | -.02 | .25^***^ | .04 | -.07 | .37^***^ | -.12^**^ | .06 | -.28^***^ | -.05 | .35^***^ | .50^***^ | .72 |  |  |  |  |
| 13. ATT Re | 4.36 | 1.39 | .81 | .08^*^ | .53^***^ | .18^***^ | .23^***^ | -.08^*^ | .27^***^ | .16^***^ | .05 | .17^***^ | .53^***^ | .71^***^ | .24^***^ | .48 |  |  |  |
| 14. SN Re ^a^ | 2.90 | 1.59 | .84 | .11^**^ | .33^***^ | .29^***^ | .17^***^ | .07 | .11^**^ | .31^***^ | .01 | .11^**^ | .58^***^ | .50^***^ | .34^***^ | .48^***^ | .72 |  |  |
| 15. PBC Re | 5.60 | 1.57 | .93 | .04 | .11^***^ | .03 | .10^*^ | -.18^***^ | .08 | .01 | .38^***^ | .06 | .03 | .05 | -.20^***^ | .06 | .09^*^ | . 81 |  |
| 16. INT Re  Items 1 & 2  Item 3 | 2.83  2.84 | 1.75  1.65 | .92 | .13^**^ | .84^***^ | .14^***^ | .17^***^ | -.08^*^ | .15^***^ | .08 | -.02 | .18^***^ | .54^***^ | .77^***^ | .30^***^ | .71^***^ | .46^***^ | .10^**^ | .79 |

The average variance extracted for constructs are presented on the principal diagonal; M = Mean; SD = Standard deviation; CR = Composite reliability; PA = Physical activity; LT = Leisure time context; Re = Recess context; PAS = Perceived autonomy support by peer; AUT = Autonomous motivation; CON = Controlled motivation; ATT = Attitude; SN = Subjective norms; PBC = Perceived behavioral control. ^a^ Values for subjective norms calculated with two observed items after omitting one of the items measuring subjective norms. **p* < .05 ***p* < .01 ****p* < .001.
