# Supplementary material for: Predicting school students’ physical activity intentions in leisure-time and school recess contexts: Testing an integrated model based on self-determination theory and theory of planned behavior
Source: PLoS One. 2021 Mar 26;16(3):e0249019. doi: 10.1371/journal.pone.0249019 (PMC7997014; doi:10.1371/journal.pone.0249019)
Supplement: S4 Table — (DOCX) [file pone.0249019.s004.docx]

**S5 Table. Results of the Multigroup Confirmatory Factor Analysis Testing Invariance of Model Parameters across Gender, Grade and School in the Leisure Time and Recess Contexts**

|  | Leisure time context | | | | | | |  | Recess context | | | | | | |
| --- | --- | --- | --- | --- | --- | --- | --- | --- | --- | --- | --- | --- | --- | --- | --- |
|  | YBχ^2^ | *df* | CFI | RMSEA | ΔSB- χ^2^ | Δ*df* | *p* |  | YBχ^2^ | *df* | CFI | RMSEA | ΔSB- χ^2^ | Δ*df* | *p* |
| Invariance test for gender |  |  |  |  |  |  |  |  |  |  |  |  |  |  |  |
| Perceived autonomy support |  |  |  |  |  |  |  |  |  |  |  |  |  |  |  |
| baseline | .000 | 0 | 1.000 | .000 | - | - | - |  | .000 | 0 | 1.000 | .000 | - | - | - |
| λ constrained | .812 | 2 | 1.000 | .000 | .812 | 2 | .666 |  | .434 | 2 | 1.000 | .000 | .434 | 2 | .805 |
| Autonomous motivation |  |  |  |  |  |  |  |  |  |  |  |  |  |  |  |
| baseline | .399 | 2 | 1.000 | .000 | - | - | - |  | .582 | 2 | 1.000 | .000 | - | - | - |
| λ constrained | 10.506 | 5 | .996 | .051 | 9.452 | 3 | .023 |  | 1.260 | 5 | 1.000 | .000 | .635 | 3 | .888 |
| Controlled motivation |  |  |  |  |  |  |  |  |  |  |  |  |  |  |  |
| baseline | 2.259 | 1 | .999 | .055 | - | - | - |  | 2.774 | 3 | 1.000 | .000 | - | - | - |
| λ constrained | 2.574 | 4 | 1.000 | .000 | .480 | 3 | .923 |  | 5.033 | 6 | 1.000 | .000 | 2.147 | 3 | .542 |
| Attitude |  |  |  |  |  |  |  |  |  |  |  |  |  |  |  |
| baseline | 3.626 | 4 | 1.000 | .000 | - | - | - |  | 3.781 | 6 | 1.000 | .000 | - | - | - |
| λ constrained | 5.148 | 8 | 1.000 | .000 | 1.644 | 4 | .801 |  | 32.159 | 10 | .983 | .072 | 26.348 | 4 | .000 |
| λ partially constrained ^a^ | - | - | - | - | - | - | - |  | 6.490 | 9 | 1.000 | .000 | 2.668 | 3 | .446 |
| Subjective norms |  |  |  |  |  |  |  |  |  |  |  |  |  |  |  |
| baseline | .000 | 0 | 1.000 | .000 | - | - | - |  | .000 | 0 | 1.000 | .000 | - | - | - |
| λ constrained | .894 | 2 | 1.000 | .000 | .894 | 2 | .640 |  | .334 | 2 | 1.000 | .000 | .334 | 2 | .846 |
| Perceived behavioural control |  |  |  |  |  |  |  |  |  |  |  |  |  |  |  |
| baseline | .000 | 0 | 1.000 | .000 | - | - | - |  | .000 | 0 | 1.000 | .000 | - | - | - |
| λ constrained | 1.428 | 2 | 1.000 | .000 | 1.428 | 2 | .490 |  | 1.008 | 2 | 1.000 | .000 | 1.008 | 2 | .604 |
| Intention |  |  |  |  |  |  |  |  |  |  |  |  |  |  |  |
| baseline | .000 | 0 | 1.000 | .000 | - | - | - |  | .000 | 0 | 1.000 | .000 | - | - | - |
| λ constrained | 21.308 | 2 | .975 | .151 | 21.308 | 2 | .000 |  | .736 | 2 | 1.000 | .000 | .736 | 2 | .692 |
| λ partially constrained ^b^ | 2.635 | 1 | .998 | .062 | 2.635 | 1 | .105 |  | - | - | - | - | - | - | - |
| Invariance test for the grades |  |  |  |  |  |  |  |  |  |  |  |  |  |  |  |
| Perceived autonomy support |  |  |  |  |  |  |  |  |  |  |  |  |  |  |  |
| baseline | .000 | 0 | 1.000 | .000 | - | - | - |  | .000 | 0 | 1.000 | .000 | - | - | - |
| λ constrained | 3.489 | 4 | 1.000 | .000 | 3.489 | 4 | .480 |  | 6.041 | 4 | .998 | .043 | 6.041 | 2 | .196 |
| Autonomous motivation |  |  |  |  |  |  |  |  |  |  |  |  |  |  |  |
| baseline | 3.586 | 3 | 1.000 | .026 | - | - | - |  | 3.185 | 4 | 1.000 | .000 | - | - | - |
| λ constrained | 16.324 | 9 | .995 | .054 | 12.364 | 6 | .054 |  | 13.051 | 10 | .997 | .033 | 10.426 | 6 | .108 |
| Controlled motivation |  |  |  |  |  |  |  |  |  |  |  |  |  |  |  |
| baseline | 9.310 | 3 | .994 | .086 | - | - | - |  | 3.399 | 5 | 1.000 | .000 | - | - | - |
| constrained | 11.194 | 9 | .998 | .029 | 3.184 | 6 | .785 |  | 14.492 | 11 | .992 | .034 | 13.238 | 6 | .039 |
| Attitude |  |  |  |  |  |  |  |  |  |  |  |  |  |  |  |
| baseline | 4.143 | 7 | 1.000 | .000 | - | - | - |  | 16.644 | 9 | .994 | .055 | - | - | - |
| λ constrained | 12.530 | 15 | 1.000 | .000 | 8.511 | 8 | .385 |  | 29.656 | 17 | .991 | .051 | 12.819 | 8 | .118 |
| Subjective norms |  |  |  |  |  |  |  |  |  |  |  |  |  |  |  |
| baseline | .007 | 2 | 1.000 | .000 | - | - | - |  | 1.478 | 2 | 1.000 | .000 | - | - | - |
| λ constrained | 4.989 | 6 | 1.000 | .000 | 4.455 | 4 | .348 |  | 3.021 | 6 | 1.000 | .000 | 1.639 | 4 | .802 |
| Perceived behavioural control |  |  |  |  |  |  |  |  |  |  |  |  |  |  |  |
| baseline | .000 | 0 | 1.000 | .000 | - | - | - |  | .239 | 1 | 1.000 | .000 | - | - | - |
| λ constrained | 2.336 | 4 | 1.000 | .000 | 2.336 | 4 | .674 |  | 9.698 | 5 | .988 | .058 | 8.875 | 4 | .064 |
| λ partially constrained ^c^ | - | - | - | - | - | - | - |  | 4.765 | 4 | .998 | .026 | 4.187 | 3 | .242 |
| Intention |  |  |  |  |  |  |  |  |  |  |  |  |  |  |  |
| baseline | .000 | 0 | 1.000 | .000 | - | - | - |  | .000 | 0 | 1.000 | .000 | - | - | - |
| λ constrained | 2.415 | 4 | 1.000 | .000 | 2.415 | 4 | .660 |  | 3.766 | 4 | 1.000 | .000 | 3.766 | 4 | .439 |
| Invariance test for the schools |  |  |  |  |  |  |  |  |  |  |  |  |  |  |  |
| Perceived autonomy support |  |  |  |  |  |  |  |  |  |  |  |  |  |  |  |
| baseline | .000 | 0 | 1.000 | .000 | - | - | - |  | .000 | 0 | 1.000 | .000 | - | - | - |
| λ constrained | 17.634 | 4 | .984 | .110 | 17.634 | 4 | .002 |  | .308 | 4 | 1.000 | .000 | .308 | 4 | .961 |
| λ partially constrained ^d^ | 3.460 | 3 | .999 | .023 | 3.460 | 3 | .326 |  | - | - | - | - | - | - | - |
| Autonomous motivation |  |  |  |  |  |  |  |  |  |  |  |  |  |  |  |
| baseline | 0.161 | 3 | 1.000 | .000 | - | - | - |  | 1.860 | 3 | 1.000 | .000 | - | - | - |
| λ constrained | 2.103 | 9 | 1.000 | .000 | 1.849 | 6 | .933 |  | 7.264 | 9 | 1.000 | .000 | 5.714 | 6 | .456 |
| Controlled motivation |  |  |  |  |  |  |  |  |  |  |  |  |  |  |  |
| baseline | 1.123 | 1 | 1.000 | .021 | - | - | - |  | 4.055 | 5 | 1.000 | .000 | - | - | - |
| λ constrained | 14.044 | 7 | .994 | .060 | 12.770 | 6 | .047 |  | 10.963 | 11 | 1.000 | .000 | 7.384 | 6 | .286 |
| Attitude |  |  |  |  |  |  |  |  |  |  |  |  |  |  |  |
| baseline | 17.777 | 9 | .993 | .059 | - | - | - |  | 18.494 | 11 | .994 | .049 | - | - | - |
| λ constrained | 30.880 | 16 | .989 | .057 | 13.228 | 7 | .067 |  | 32.619 | 19 | .990 | .050 | 14.139 | 8 | .078 |
| Subjective norms |  |  |  |  |  |  |  |  |  |  |  |  |  |  |  |
| baseline | .946 | 2 | 1.000 | .000 | - | - | - |  | .415 | 1 | 1.000 | .000 | - | - | - |
| λ constrained | 10.395 | 6 | .988 | .051 | 9.778 | 4 | .044 |  | 9.947 | 5 | .989 | .059 | 9.141 | 4 | .058 |
| Perceived behavioural control |  |  |  |  |  |  |  |  |  |  |  |  |  |  |  |
| baseline | .000 | 0 | 1.000 | .000 | - | - | - |  | .000 | 0 | 1.000 | .000 | - | - | - |
| λ constrained | 3.130 | 4 | 1.000 | .000 | 3.130 | 4 | .536 |  | 11.756 | 4 | .980 | .083 | 11.756 | 4 | .019 |
| λ partially constrained ^e^ | - | - | - | - | - | - | - |  | 2.092 | 3 | 1.000 | .000 | 2.092 | 3 | .554 |
| Intention |  |  |  |  |  |  |  |  |  |  |  |  |  |  |  |
| baseline | .000 | 0 | 1.000 | .000 | - | - | - |  | .000 | 0 | 1.000 | .000 | - | - | - |
| λ constrained | 7.007 | 4 | .996 | .052 | 7.007 | 4 | .136 |  | 2.214 | 4 | 1.000 | .000 | 2.214 | 4 | .697 |

YBχ^2^ = Yuan-Bentler scaled chi-square value of model fit; *df* = Degrees of freedom for chi-square statistic; CFI = Comparative fit index; RMSEA = Root mean square error of approximation; ΔSBχ^2^ = Incremental change in Satorra-Bentler scaled chi-square value; Δ*df* = Incremental change in degrees of freedom; *p* = probability of the ΔSBχ^2^; λ = factor loading; ^a^Factor loading of one attitude item (Att3, see S2 Table) item for the recess context was non-invariant across gender; ^b^Factor loading of one intention item (Int1, see S1 Table) for the leisure-time context was non-invariant across gender; ^c^Factor loading of one perceived behavioral control item (Pbc2, see S2 Table) for the recess contexts was non-invariant in the 7^th^ grade subsample; ^d^Factor loading of one perceived autonomy support parceled item (PasA, see S1 Table) item for the leisure-time context was non-invariant in the School B subsample; ^e^Factor loading of one perceived behavioral control item (Pbc2, see S2 Table) for the recess context was non-invariant across the School B subsample
