# Supplementary material for: Predicting school students’ physical activity intentions in leisure-time and school recess contexts: Testing an integrated model based on self-determination theory and theory of planned behavior
Source: PLoS One. 2021 Mar 26;16(3):e0249019. doi: 10.1371/journal.pone.0249019 (PMC7997014; doi:10.1371/journal.pone.0249019)
Supplement: S5 Table — (DOCX) [file pone.0249019.s005.docx]

**S6 Table. Goodness-of-Fit Statistics for Single-Group Structural Equation Models for Full Sample, and Gender, Grade and School Sub-Samples in the Leisure Time and Recess Contexts**

|  | Leisure Time | | | |  | Recess | | | |
| --- | --- | --- | --- | --- | --- | --- | --- | --- | --- |
|  | YBχ^2^ | *df* | CFI | RMSEA |  | YBχ^2^ | *df* | CFI | RMSEA |
| Full sample (N = 845) | 610.891 | 226 | .965 | .045 |  | 504.278 | 228 | .978 | .038 |
| Gender |  |  |  |  |  |  |  |  |  |
| Girls (*n* = 457) | 464.821 | 228 | .960 | .048 |  | 427.933 | 231 | .970 | .043 |
| Boys (*n* = 388) | 508.078 | 230 | .949 | .056 |  | 417.663 | 229 | .970 | .046 |
| Grade |  |  |  |  |  |  |  |  |  |
| Grade 7 (*n* = 292) | 467.796 | 230 | .940 | .060 |  | 337.391 | 229 | .975 | .040 |
| Grade 8 (*n* = 259) | 413.981 | 229 | .950 | .056 |  | 391.752 | 233 | .958 | .051 |
| Grade 9 (*n* = 294) | 437.749 | 229 | .950 | .056 |  | 426.516 | 232 | .961 | .053 |
| School |  |  |  |  |  |  |  |  |  |
| School A (*n* = 325) | 409.725 | 228 | .959 | .050 |  | 360.242 | 232 | .974 | .041 |
| School B (*n* = 274) | 410.960 | 229 | .954 | .054 |  | 391.245 | 231 | .962 | .050 |
| School C (*n* = 246) | 420.876 | 229 | .945 | .058 |  | 353.115 | 229 | .970 | .047 |

Note. YBχ^2^ = Yuan-Bentler scaled chi-square value of model fit; df = Degrees of freedom for chi-square statistic; CFI = Comparative fit index; RMSEA = Root mean square error of approximation.
