# Supplementary material for: Predicting school students’ physical activity intentions in leisure-time and school recess contexts: Testing an integrated model based on self-determination theory and theory of planned behavior
Source: PLoS One. 2021 Mar 26;16(3):e0249019. doi: 10.1371/journal.pone.0249019 (PMC7997014; doi:10.1371/journal.pone.0249019)
Supplement: S1 Appendix — (DOCX) [file pone.0249019.s006.docx]

**S3 Appendix**

**Parceling Process Applied for Autonomous Motivation, Controlled Motivation, and Perceived Autonomy Support by Peers Constructs**

Four observed variables measuring the each form of motivation from self-determination theory, namely intrinsic, identified, introjected, extrinsic, were parceled randomly into two averaged variables. The two parceled variables for intrinsic (ImA, ImB) and identified (IdA, IdB) regulation were used to indicate the autonomous motivation latent variable in both contexts (AUT_LT, AUT_RE). The two parceled variables measuring introjected regulation (IjA, IjB) and extrinsic motivation (ExA, ExB) were used to indicate the controlled motivation latent variable in both contexts (CON_LT, CON_RE). The latent autonomous and controlled motivation variables were, therefore, each indicated by four parceled items formed from eight observed items. The parceled items were used in subsequent confirmatory factor analyses (CFA) and structural equation models. Composite reliabilities (CR) for the autonomous motivation construct using all eight observed items (leisure time CR = .90; recess CR = .93) and using the four parceled items (leisure time, CR = .86; recess, CR = .91) were satisfactory. Reliabilities for the controlled motivation construct using all eight items (leisure time, CR = .83; school, CR =.92) and the four parceled items (leisure time, CR = .80; recess, CR = .91) were also satisfactory.

One item with a factor loading falling well short of the .70 criterion was deleted from the perceived autonomy support by peers construct for the leisure time (“I am able to talk with me friends about the sports and/or exercises I do in my free time”, λ = .55) and recess (“ I am able to talk with me friends about the physical activities I participate during the recesses”, λ = .52) contexts. The deleted items focused on activity in while communication with a friend, which may have deviated from the other items that focused on personal support, for example receiving feedback or help. The range of the loadings of the remaining items was λ = .67 to .88 and λ = .67 to .89 in the leisure time and the recess contexts, respectively. The remaining six items were randomly parceled into three mean variables (PasA, PasB, PasC), for the both contexts separately. These three parceled items were used in subsequent multigroup CFA and structural equation modeling analyses to indicate the perceived autonomy support by peers latent variable, for the both contexts separately. For the full sample, the composite reliabilities for the scales with all seven observed items (leisure time, CR = .89; recess, CR = .92), with the six observed items (leisure time, CR = .89; recess, CR = .93) and with the three parceled items (leisure time, CR= .89; recess, CR = .93) were satisfactory.
